# How EU policies could reduce nutrient pollution in European inland and coastal waters

**DOI:** 10.1016/j.gloenvcha.2021.102281

**Published:** 2021-07

**Authors:** B. Grizzetti, O. Vigiak, A. Udias, A. Aloe, M. Zanni, F. Bouraoui, A. Pistocchi, C. Dorati, R. Friedland, A. De Roo, C. Benitez Sanz, A. Leip, M. Bielza

**Affiliations:** a European Commission Joint Research Centre (JRC), Ispra, VA, Italy; b Arhs Developments, Italy; c Emgrisa, Madrid, Spain; d Seidor, Barcelona, Spain

**Keywords:** Nutrient pollution, Policy scenarios, EU Water Framework Directive, EU Marine Strategy Framework Directive

## Abstract

Intensive agriculture and densely populated areas represent major sources of nutrient pollution for European inland and coastal waters, altering the aquatic ecosystems and affecting their capacity to provide ecosystem services and support economic activities. Ambitious water policies are in place in the European Union (EU) for protecting and restoring aquatic ecosystems under the Water Framework Directive and the Marine Strategy Framework Directive. This research quantified the current pressures of point and diffuse nitrogen and phosphorus emissions to European fresh and coastal waters (2005–2012), and analysed the effects of three policy scenarios of nutrient reduction: 1) the application of measures currently planned in the Rural Development Programmes and under the Urban Waste Water Treatment Directive (UWWTD); 2) the full implementation of the UWWTD and the absence of derogations in the Nitrates Directive; 3) high reduction of nutrient, using best technologies in wastewaters treatment and optimal fertilisation in agriculture. The results of the study show that for the period 2005–2012, the nitrogen load to European seas was 3.3–4.1 TgN/y and the phosphorus load was 0.26–0.30 TgP/y. Policy measures supporting technological improvements (third scenario) could decrease the nutrient export to the seas up to 14% for nitrogen and 20% for phosphorus, improving the ecological status of rivers and lakes, but widening the nutrient imbalance in coastal ecosystems (i.e. increasing nitrogen availability with respect to phosphorus), affecting eutrophication. Further nutrient reductions could be possible by a combination of measures especially in the agricultural sector. However, without tackling current agricultural production and consumption system, the reduction might not be sufficient for achieving the goals of EU water policy in some regions. The study analysed the expected changes and the source contribution in different European regional seas, and highlights the advantages of addressing the land-sea dynamics, checking the coherence of measures taken under different policies.

## Highlights

• In EU nutrient pollution harms aquatic ecosystems condition and their services.
• Current nutrient load to European seas is 3.3–4.1 TgN/y and 0.26–0.30 TgP/y.
• EU policy measures could decrease the nutrient export to the seas −14% N and −20% P.
• Widening the nutrient imbalance in coastal ecosystems, affecting eutrophication.
• Further nutrient reductions could be possible by a combination of measures.

## 1 Introduction

In Europe, intensive agricultural practices together with high population density represent important sources of nutrients for fresh and coastal waters (Sutton et al., 2011). Nutrient pollution is one of the major pressures on European aquatic ecosystems altering their condition (Grizzetti et al., 2017). At present in the EU more than half of water bodies are not in good ecological status, as required by the EU Water Framework Directive (2000/60/EC), with nutrient being one of the major causes of degradation (Poikane et al., 2019a). Many marine ecosystems suffer from hypoxia and eutrophication (Diaz and Rosenberg, 2008, Romero et al., 2013). In estuaries and coastal waters the increase of nutrient availability from riverine loads foster primary productivity causing the phenomenon of eutrophication (Howarth et al., 2011). In particular, the imbalance of nitrogen and phosphorus over silica can be responsible for the proliferation of harmful algal blooms (HABs) (Billen and Garnier, 2007). Eutrophication impairs water quality and alters the condition and functioning of fresh and marine ecosystems, affecting their capacity to supply key ecosystem services and sustain economic activities, such as water purification, coastal protection, lifecycle maintenance, drinking water, fishing, shellfish farming, recreation and tourism (Culhane et al., 2019, Grizzetti et al., 2016, Liquete et al., 2016, Piroddi et al., 2017).

Ambitious water policies are in place in the European Union (EU) for protecting and restoring freshwater and marine ecosystems under the Water Framework Directive (WFD, 2000/60/EC) and the Marine Strategy Framework Directive (MFSD, 2008/56/EC). The goal of the WFD is to achieve Good Ecological Status for all water bodies in the EU, including river, lakes, transitional and coastal waters. Under the WFD, Member States develop River Basin Management Plans (RBMP) for the protection and restoration of aquatic ecosystems and the sustainable use of water resources. Measures adopted under the Nitrates Directive (91/676/EEC) and the Common Agricultural Policy (CAP), to reduce nutrient water pollution from agriculture, as well as actions taken under the Urban Waste Water Treatment Directive (UWWTD, 91/271/EEC) contribute to the objectives of the WFD. Similarly, the MSFD aims at achieving Good Environmental Status (GES) of all EU marine waters, protecting biodiversity and resilience of marine ecosystems, and promoting their sustainable use. The GES is described by 11 qualitative descriptors and nutrient pollution directly or indirectly influences several of them concerning biodiversity, non-indigenous species, fish population, reproduction, eutrophication and sea floor integrity (Descriptors 1–6). European seas are also protected under four international Conventions: the Helsinki Convention on the Baltic Sea (HELCOM, 2020), the OSPAR Convention on the North-East Atlantic (OSPAR, 2020), the Barcelona Convention on the Mediterranean (UNEP, 2020) and the Bucharest Convention on the Black Sea (Black Sea Commission, 2020).

The provision of ecosystems services depends on the condition of aquatic ecosystems (Grizzetti et al., 2019). Current pressures and future changes, such as climate changes, can further degrade the status of aquatic ecosystems (Jonkers et al., 2019, Lajaunie-Salla et al., 2018, Macias et al., 2018) and the resilience of water resources (Zampieri et al., 2019). The assessment of the impact of EU water policy on water quality and freshwater and coastal ecosystems requires the understanding of the sources and sinks of nutrients along the land-sea continuum at the continental scale. Modelling tools can be useful to study the impacts of future scenarios, policy measures and climate changes at the regional and continental scale (Arheimer et al., 2012, Bartosova et al., 2019, Bouraoui et al., 2014, Ludwig et al., 2010, Seitzinger et al., 2010), and to check the coherence of different policy targets, for instance between water (WFD and MFSD) and agricultural (Common Agricultural Policy, CAP) policies.

Several models have been developed to estimate riverine nutrient load from land to the sea at the global scale, such as Global NEWS (Mayorga et al., 2010, Seitzinger et al., 2005) and IMAGE-GNM (Beusen et al., 2016, Beusen et al., 2015) with spatial resolution of 0.5⁰ grid cells, and at the continental scale, such as E-HYPE (Donnelly et al., 2013, Lindström et al., 2010) and GREEN (Grizzetti et al., 2012) with spatial resolution of catchments around 200 km^2^, and have also been applied for scenario analysis (Blaas and Kroeze, 2016, Seitzinger et al., 2005). However, the assessment of policy scenarios specifically for the European Union requires a straightforward and at the same time spatially detailed analysis that makes use of consistent data across many different countries, accounts for the wide climatic, hydrological and socio-economic gradients, and links local pressures, freshwater bodies, and coastal waters.

To address these specific needs, the objective of this study was to quantify the current nitrogen and phosphorus pressures on European fresh and coastal waters and to assess the effects of policy scenarios to reduce nutrient pollution with progressive levels of ambition. In particular, the study aimed at developing a spatial analysis integrating inland and coastal waters to fit different level of policy intervention from the water body to the river basin and the regional sea, including the most recent data available across European countries, reported under EU legislation obligations.

Key questions of the analysis were: ‘Would the current measures in place ensure nutrient concentration in surface water and loads to the seas in line with the goals of the EU water policy (WFD and MSFD)?’ and ‘How far different scenarios of nutrient pressures reduction could contribute to the improvements of ecological status of freshwaters and the reduction of nutrient export to the sea?’ To address these questions we applied a new version of the model GREEN (Grizzetti et al., 2012), which has a much higher spatial resolution (catchments of around 10 km^2^) and updated spatial information on nutrient sources. The model GREEN has been used in previous studies for assessing the nutrient loads to the European seas, the nitrogen retention in European freshwaters, and for scenario analysis (Bouraoui et al., 2014, Grizzetti et al., 2012, La Notte et al., 2017, Leip et al., 2015, Malagó et al., 2019).

## 2 Materials and methods

### 2.1 Overview of the assessment

The model GREEN (Geospatial Regression Equation for European Nutrient losses) (Grizzetti et al., 2012, Grizzetti et al., 2008) was applied to assess the nitrogen and phosphorus pressures on European fresh and coastal waters. In specific, nutrients’ annual load and average concentration at different points of the river network and at the river outlets to the sea were estimated, as well as the contribution of different diffuse and point sources to the total load.

**Fig. 1:**
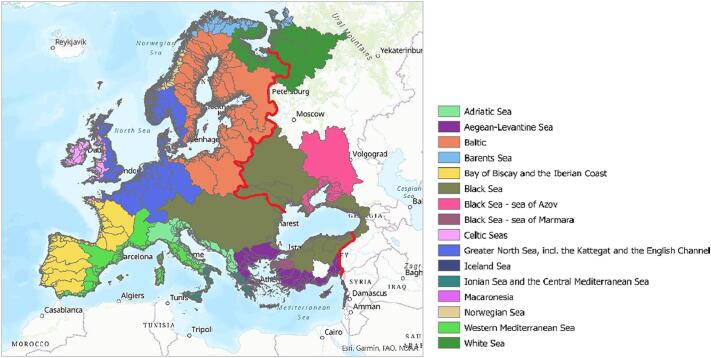
Extent covered by the analysis: European river basins up to the red line. Colours indicate the river basins per European Regional Seas. (For interpretation of the references to colour in this figure legend, the reader is referred to the web version of this article.)

The spatial extent of the analysis covered all river basins draining into European seas and whose waters fall completely or in part within EU countries (EU 28 countries as on 1st January 2020) (Fig. 1). EU overseas territories were not included in the study. The assessment was carried out for the period 2005–2012 (with a focus on 2012) for which best available data for model input and calibration were available at the European scale.

Nitrogen and phosphorus inputs from different sources in river basins were estimated for all Europe to describe the current nutrient pressures on waters (reference scenario). Then, three scenarios were developed to simulate the effect of policies with progressive levels of ambition to reduce nutrient pressures (see Section 2.5). The scenarios on nutrients were associated to concurrent measures to alleviate water scarcity (De Roo et al., 2020).

Finally, the results of the scenarios were analysed in view of the EU water policy (Water Framework Directive) target of good ecological status for all rivers, lakes and transitional waters in the EU and the good environmental status for marine waters.

### 2.2 The model GREEN

The model GREEN (Grizzetti et al., 2012) includes a geospatial data model for Europe (geo-database), where data are linked to the hydrological structure of the river network (developed in ESRI environment), and routines to model nitrogen and phosphorus flow in the river basin according to different pathways (developed in SQL server environment).

The geospatial data model is based on the CCM2 model (Vogt et al., 2007) and includes the Ecrin data for lakes (EEA, 2012). Europe is divided in ~ 1 million catchments of 7 km^2^ average size. Each catchment has an elementary river stretch, except in coastal areas, where river stretch might be absent.

The model distinguishes two major pathways to represent the fate of nutrients: diffuse sources that undergo a retention in the land phase (basin retention) before reaching the stream, and point sources that are directly discharged into surface waters. Once in the river all sources are reduced by the in-stream retention (river retention). Diffuse sources include nutrient from mineral fertilisers, manure application, nitrogen crop and soil fixation, nitrogen atmospheric deposition, background losses (only for phosphorus) and inputs from scattered dwellings, i.e. isolated houses that are not connected to sewerage systems. Point sources consist of urban and industrial wastewater discharges. Nutrient input from the different sources and the basin and river retention are simulated in each small catchment and routed through the river network. Basin retention is modelled as a decay function proportional to the inverse of the total annual precipitation in the catchment; river retention is estimated as a decay function proportional to the river length, considered as a proxy for water residence time. In addition, lake retention is simulated as a function of lakes residence time and average depth. Two parameters are calibrated at the European scale, a basin retention coefficient (basinCoeff) and a river retention coefficient (riverCoeff). In the model, inputs from agricultural sources are reduced by the basin and then the river retention, while point sources are reduced only by the river retention. Input from scattered dwellings is considered to be halved before reaching the stream. Nitrogen atmospheric deposition and phosphorus background losses are split into two parts, i.e. inputs to agricultural land undergo the basin retention (which include also the crop uptake), while in all other areas they are reduced by a fixed rate, derived from the literature, before entering into the stream (Model equations are provided in SuppMat S1).

Model input and output data are organised in the geo-database at the spatial resolution of catchments. The model results are aggregated per Functional Elementary Catchments (FECs) (EEA, 2012), containing on average 2–3 catchments), per river basins and River Basin Districts (RBD), according to the WFD (EEA, 2019a, EEA, 2019), and per basins draining into marine regions, according to the MFSD (EEA, 2018a).

For the present study a new version of the model GREEN was developed. The major changes from the previous version (Grizzetti et al., 2012) are the higher spatial resolution of the model application (sub-catchments of 7 km^2^ average size, 180 km^2^ in the previous version), the inclusion of all coastal areas (also in absence of streams), the adoption of Corine Land Cover maps for land cover allocation, and a new setup of nutrient input data corresponding to the most recent European data publicly available. In addition, the model was calibrated using a different dataset and for a more recent period (2008–2012).

### 2.3 Input of nutrient sources and model parameterisation

The allocation of different land cover type in each catchment was based on the Corine Land Cover map (CLC, 2012) (grid at 100 m resolution, available for year 2000, 2006 and 2012 for Europe). For areas falling outside the CLC, land cover data was taken from the Climate Change Initiative Land Cover map (ESA, 2017) (global grid at 300 m resolution, yearly maps from 1992 to 2015). Time series on total agricultural area and fertiliser application per major crops and grassland were provided by the model CAPRI at the administrative unit level NUTS2 (European Commission, 2018) (yearly values from 1984 to 2013, estimated considering year 2012 as base year). The CAPRI model covers EU28, Norway, and non-EU countries in the Balkans. Information on fertiliser applications were also retrieved from FAO (FAOSTAT, 2020), for European countries not covered by CAPRI.

Two annual time series of nitrogen and phosphorus mineral and manure fertiliser maps from 1995 to 2015 were developed for Europe based on two different methods. The first adopts the CLC for the spatial location of crops and the CAPRI model for the information on fertilisers and utilised agricultural area by crop type. The second considers the extent and location of agricultural area provided by ESA and the total fertilisation rate reported by FAO per country. The first approach offers higher details on crop location and fertilisation, and the utilised agricultural area is consistent with data reported by national statistics of EU28 countries (as CAPRI is based on EUROSTAT). The second method provides average nutrient fertilisation rates per country (without distinguishing crop types) in agricultural areas detected by satellite images, and can be applied to all European countries (also those not covered by CAPRI) and at the global scale. In order to develop nitrogen and phosphorus fertiliser maps for all river basins draining in European seas the results of the two methods were combined, completing the areas (countries) not covered by the first method by the second one. The model CAPRI provides also the nitrogen fixation by crops. This information was combined with crop location to establish time series maps of nitrogen crop fixation. In addition nitrogen fixation in soils of 5 kgN/ha and phosphorus background sources (originated from rock weathering and atmospheric deposition) of 0.15 kgP/ha were considered as in the previous version of the model. Annual total nitrogen atmospheric deposition was computed using the data from the model EMEP (EMEP, 2020).

Nitrogen and phosphorus inputs from domestic waste were based on the most recent information reported by EU countries under the Urban Waste Water Treatment Directive (Vigiak et al., 2020). Further, industrial discharges were retrieved from the European Pollutant Release and Transfer Register (E-PRTR) (EEA, 2018b, EEA, 2018) and official statistics available in EUROSTAT and WHO for non EU countries. The dataset is based on the spatial analysis of different sources and pathways of human and industrial waste to surface waters, including the location and level of treatment of each treatment plants (Vigiak et al., 2019). While annual time series were available for nutrient inputs from agriculture and nitrogen atmospheric deposition, inputs from scattered dwellings and point sources discharges were available only for the year 2012 and applied as constant for the period 2005–2012.

In each catchment the stream length was based on the CCM2 model and the time series of annual precipitation was computed from EFAS-Meteo (Arnal et al., 2019, Ntegeka et al., 2013). The water flow estimated by the model LISFLOOD (De Roo et al., 2020), which comprised water abstractions and irrigation, was included in the analysis, by interpolating the average annual water flow, provided by the model on a 1 km^2^ grid resolution, at each catchment outlet of the geospatial data model.

### 2.4 Model calibration and validation

The calibration of the two model parameters (basinCoeff and riverCoeff) was performed over the period 2008–2012 using the data publicly available in the Waterbase (EEA, 2016) on mean annual total nitrogen and total phosphorus concentrations. In total 9335 observations for total nitrogen and 13,890 observations for total phosphorus were used for the calibration, corresponding to 3685 and 4163 different locations in Europe for nitrogen and phosphorus, respectively (additional information in SuppMat S2). The period 2008–2012 was selected for the calibration as the quality of the data and their spatial coverage (despite not homogeneous across Europe) were better than in previous years. In addition, using the whole period for calibration was necessary to cover different hydrological years (in the studied area the average annual rainfall in the period 2008–2012 ranged between 739 and 881 mm). The annual total nitrogen and total phosphorus “observed” loads used for calibration were computed multiplying the concentrations times the annual water flow estimated by the model LISFLOOD for all the points in the river network where observed data were available.

The calibration was carried out using a routine designed specifically for this purpose using R (R Core Team, 2014), including several optimization algorithms (Simulated annealing, Genetic Algorithm, etc.). It was found that the application of a Markov Chain Monte Carlo method (Kuczera and Parent, 1998) was the most efficient when the number of evaluations performed in the calibration process was<500 (which is desirable for practical purposes given the computation time requirements for each evaluation). Several goodness of fit measures were considered for the optimal parameter selection, among others: Nash-Sutcliffe Efficiency (NSE), Percent Bias (pbias), Relative Index of Agreement (rd), Relative Nash-Sutcliffe efficiency (rNSE) (Krause et al., 2005). The calibration was run both with actual values and log10 values to compare the effects of large nutrient loads (generally in downstream catchments) with small loads (generally in upstream catchments). The final parameters selection was based on the Nash-Sutcliffe Efficiency (NSE) (Nash and Sutcliffe, 1970) statistic computed on the no-log simulation.

The model was run from 2005 to 2012. The verification of the model estimation was conducted comparing the nutrients load estimated by the model GREEN at the outlet of the 50 largest rivers in Europe with data available from various independent sources in the literature. Correlation was computed for the whole data set as well as for the different data sources. The linear fit between reported and modelled freshwater runoff and nutrient loads was computed, assuming the intersect at 0.

### 2.5 Scenarios development

The scenarios of reduction of nutrient pollution in inland and coastal waters were designed with the specific intent of providing support to the EU water policy and with a progressive increasing level of ambition. Three scenarios were developed, including both a reduction of urban point sources and diffuse agricultural pollution (Table 1). The first scenario, called Business As Usual Scenario (BAU), was meant to represent the current level of investment in water protection from nutrient pollution foreseen by actual planning instruments in EU Member States, notably, investments in upgrading urban waste water treatment plants under the UWWTD Art.17 (Benitez Sanz et al., 2018), and investments in measures to protect water quality under the Rural Development Programme (RDP) 4b priority. In the latter, the effectiveness of the mix of measures was assumed to be 10% reduction of the nitrogen and phosphorus entering the water system (Sarteel et al., 2016). This reduction was applied in all catchments, but only considering the fraction of Utilised Agricultural Area (UAA) that is covered by RDP 4b priority in the NUTS2, as the location of UAA subject to RDP 4b priority is unknown. The second scenario, called Nutrient Scenario (NUTR), intended to represent a full or enhanced implementation of two EU Directives, the UWWTD for collecting and treating wastewater from urban agglomerations, and the Nitrates Directive to protect freshwater from agricultural pollution. In the NUTR scenario all wastewater treatments in the EU were set compliant with the requirement of the UWWTD (Pistocchi et al., 2019), introducing emission limits for agglomerations with more than 2000 population equivalent, such as biological carbon removal and, for agglomerations discharging in areas prone to eutrophication, also nutrient removal. In the NUTR scenario nitrogen application in agricultural fields was limited to maximum 170 kgN/ha in all Nitrates Vulnerable Zones in the EU irrespectively of the presence of Derogations (i.e. specific areas under particular conditions where the application of nitrogen from manure can be higher than the maximum limits, https://ec.europa.eu/environment/water/water-nitrates, accessed in November 2020) (phosphorus in manure application was reduced proportionally). Finally the third scenario, called Maximum Technically Feasible Reduction scenario (MTFR), considered that all wastewaters in the EU are treated with the maximum level of nutrient reduction currently possible (correspondent to a tertiary treatment with an enhanced reduction of phosphorus), while mineral fertiliser are reduced according to an optimal fertilisation. To estimate the optimal mineral nitrogen fertilisation, first the total optimal crop nitrogen requirement per country was computed keeping the nitrogen surplus (difference between nitrogen total inputs and total outputs to the soil, gross nitrogen balance data from EUROSTAT, year 2012) to a minimum, which was set to 10% of the output. Then, the optimal mineral nitrogen fertilisation per country was estimated as difference between the total optimal crop nitrogen requirements, the other nitrogen inputs (from atmospheric deposition and crop and soil fixation) and 70% of the manure nitrogen application, i.e. considering that the efficiency of crops to assimilate nitrogen from manure is 70% (Webb et al., 2013). Finally, the country specific reduction of mineral nitrogen fertiliser was obtained distributing the reduction rate according to a weight that accounts for catchments with higher application of mineral + manure fertilisers. The corresponding reduction of mineral phosphorus was estimated. In the MTFR scenario the optimal fertilisation was simulated reducing mineral nitrogen fertiliser without changing the manure nitrogen applications. This means that in region with high livestock density the mineral fertilisation is reduced in the scenario but the total amount of nitrogen input to the soil can still be elevated. In the MTFR scenario the average Nitrogen Use Efficiency (defined as the ratio between nitrogen output and nitrogen input) per EU countries is 0.75 compared to 0.58 in the REF scenario.

**Table 1:** Description of the scenarios. The scenarios for water quantity are described in De Roo et al. (2020).

Scenario acronym	**Water quantity (model LISFLOOD)**	**Water quality – Point pollution (model GREEN)**	**Water quality – Diffuse pollution (model GREEN)**
**REF**	Situation in 2005–2012	Situation in 2012	Situation in 2005–2012
**BAU(Business As Usual)**	Change in irrigation efficiency, urban water usage efficiency, water re-use, and water use changes due to energy demand changes, under the current investments in River Basin Management Plants of the WFD	Nutrient reductions related to the current investments in EU under the Art.17 of the Urban Waste Water Treatment Directive (Council Directive 91/271/EEC).	Nutrient reductions due to the measures funded under the Rural Development Programme 4b priority (spatial information on the measures for implementing the Nitrates Directive were not available).
**NUTR (Nutrient Reduction)**	As BAU	Full implementation of the Urban Waste Water Treatment Directive (Council Directive 91/271/EEC) in EU.	Maximum application of manure nitrogen is limited to 170 kgN/ha in areas draining the Nitrates Vulnerable Zones (NVZ), irrespective of the presence of Derogations. The corresponding decrease of phosphorus in manure application is considered.
**MTFR (High Technically Feasible Reduction)**	Change in irrigation efficiency, urban water usage efficiency, water re-use, and water use changes due to energy demand changes under the Maximum Technical Feasibility scenario	All urban waste water treatment plants are upgraded to the highest treatment level.	Scenario of optimal fertilization. The nitrogen surplus on agricultural soils is set to 10% of the reported output. The corresponding reduction of mineral phosphorus is considered.

Each scenario of nutrient reduction was run including a corresponding scenario of measures for preventing water scarcity in the hydrological model LISFLOOD (De Roo et al., 2020). The BAU and NUTR scenarios were associated with the implementation of measures on water quantity (increase of water use efficiency in irrigation and in domestic usage, changing cooling water requirements due to change in energy demand and energy mix, and implementation of wastewater re-use for irrigation), corresponding to the level of current investments foreseen in River Basin Management Plans of the WFD. Differently, the MTFR scenario was associated to a higher level of implementation of the same measures.

### 2.6 Scenarios analysis and link to ecological targets

The scenarios were applied retrospectively to the year 2012, which was representative of average hydrological conditions over the period of analysis (average rainfall of 823 mm, compared to the range of 739–881 mm for the period 2008–2012) and for which the most accurate data were available (see Section 2.1). Annual nutrient load to the sea and concentration in freshwater estimated by the model GREEN for the three scenarios (BAU, NUTR, MTFR) were compared with the values of the reference scenario (REF, i.e. the historical situation for year 2012). The analysis was conducted at different levels of aggregation: 1) Europe (extent covered by the modelling), 2) MSFD regional sea basins, and 3) river basins, which are of interest for the WFD, as River Basin Districts are composed of one or several river basins (results not shown). The share of different nutrient input sources to the river basin was evaluated as well as their contribution to the total load in rivers. In addition, the predicted changes in nutrient concentration and N:P ratio were analysed.

The potential impact of the three scenarios on achieving the target of good ecological status established by the WFD was assessed by spatially linking the model predictions with the location and ecological conditions of water bodies reported by the EU Member States under the WFD. For this purpose, the most recent data on the ecological status of lakes, rivers and transitional waters, referring to second River Basin Management Plans, 2010/2016 (EEA, 2019b), were overlaid with the geospatial data model of GREEN. The WISE dataset includes information on water bodies’ delineation, and on the ecological status and main impact types observed. Each WISE water body was assigned to one or multiple catchments in the GREEN geospatial model according to its location and spatial geometry. A one to one correspondence between WISE water bodies and the GREEN geospatial model was not possible as the delineation of water bodies depends on decisions of River Basin District Authorities in the different EU countries, while the catchments and river network delineation in the GREEN geospatial model are derived from consistent topographic information at the European scale (CCM2 model). The water bodies area (for lakes) and length (for rivers and transitional waters) falling in each catchment of the geospatial model were computed, as one water body could lays in one or many catchments. The nutrient concentration estimated by the model GREEN for each WISE water body was computed as area-weighted or length-weighted average across the CCM2 catchments involved.

The spatial association between WISE water bodies and CCM2 catchments allowed to compute nutrient concentration estimated by the model GREEN for the WISE water bodies, and derive average nitrogen and phosphorus concentration per ecological status class and water body type (as for each water body WISE contains the information on the ecological status class, namely High, Good, Moderate, Poor or Bad status). From this analysis, the values of 2 mgN/l and 0.1 mgP/l were found consistent with average nitrogen and phosphorus concentration in water bodies in Good Ecological Status and were used as thresholds only for comparing the effects of the three different scenarios. These thresholds were computed for rivers, lakes, and transitional waters bodies considering (i) the whole EU and (ii) a subset of water bodies where ‘Nutrient Pollution’ impact was reported (25% of the total water bodies). The potential impacts of the scenarios on the ecological status was evaluated considering the percentage of lakes area and rivers length that was below the threshold in the different scenarios.

## 3 Results

### 3.1 Model calibration and validation

The results of model calibration indicated a good agreement between observed and estimated nutrient loads, with NSE of 0.96 and 0.75 for nitrogen and phosphorus, respectively (SuppMat S3).

**Fig. 2:**
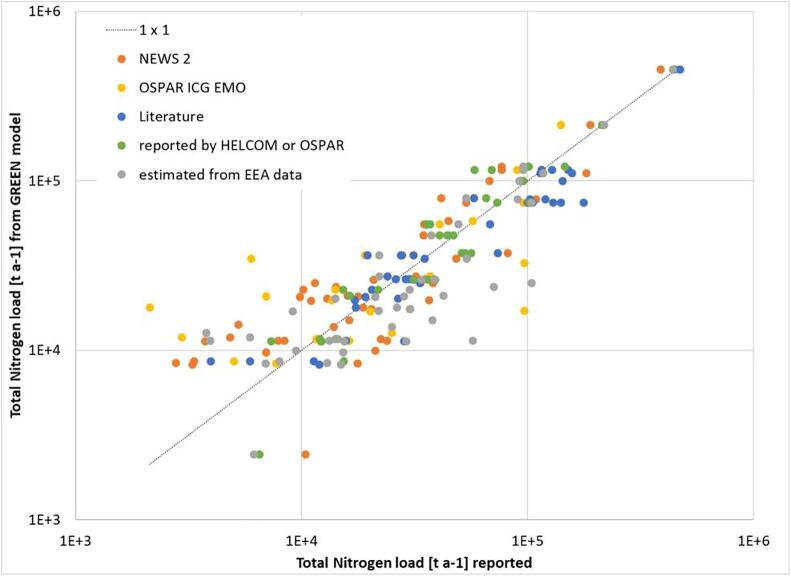
Comparison between annual Total Nitrogen loads reported by different sources and estimated by the GREEN model (tN/y) shown on a log–log scale. The sources of reported values are indicated by the dot colours and are detailed in SuppMat S4). (For interpretation of the references to colour in this figure legend, the reader is referred to the web version of this article.)

The freshwater runoff estimated by the LISFLOOD model and total nutrient loads estimated by the model GREEN at the outlet of the 50 largest European rivers showed a good agreement with the values reported by other data sources and the literature (Fig. 2 and SuppMat S4). Correlation coefficients of model estimates with loads reported by HELCOM and OSPAR Conventions are 0.85 for nitrogen and 0.90 for phosphorus; the correlation with data found in the literature (listed in SuppMat S4) is 0.94 for nitrogen and 0.84 for phosphorus. The gradients of the linear fitting are almost all around 1, indicating a good fit between reported and modelled values. Overall, the model results with respect to the nitrogen loads are better than for the phosphorus ones. Beside the good overall agreement, for some individual rivers severe deviations occur, e.g. freshwater runoff of river Scheldt is two magnitudes below the reported values, while nutrient loads fit well. On the other hand, for some rivers (e.g. Thames, Humber) GREEN provides the nutrient loads directly at the outlet to the sea, while observations are collected upstream.

### 3.2 Scenarios analysis

#### 3.2.1 Changes in nutrient inputs to river basins

In Europe (region covered by the study) for the reference year 2012 the total input of nitrogen was estimated at 27.6 TgN (23.3 TgN in EU28) of which 71% from mineral and manure fertilisers, 17% from atmospheric deposition, 8% from biological natural fixation (in crops and soils), 3.5% from human and industrial waste waters and 0.5% from scattered dwellings. The total input of phosphorus was estimated at 4.1 TgP (3.5 TgP in EU28), of which 93% from mineral and manure fertilisers, 5% from human and industrial waste waters, 2.3% from natural background and 0.5% from scattered dwellings. On average point nutrient sources represented only 3–5% of the total input to the river basin system, but locally they could reach much higher shares.

Overall, in the BAU, NUTR and MTFR scenario compared to the REF scenario, the total nitrogen input was reduced by 1%, 5% and 18% respectively, and the total phosphorus input was reduced by 1%, 10% and 14%, respectively (SuppMat S5). In particular, for EU28 the MTFR scenario foresaw a reduction of 21% of nitrogen input (-24% point sources and −25% agricultural sources) and 16% of phosphorus input (-49% point sources and −16% agricultural sources) (Tables S5.3 and S5.4 in SuppMat). When looking at regional differences, the BAU scenario resulted in limited reduction (1–2%) in all regions for both nitrogen and phosphorus, while the NUTR scenario involved large changes in some regions, such as in the basins draining into the Greater North Sea characterised by intensive agriculture and livestock production. The MTFR scenario foresaw reductions between 13 and 37% for total nitrogen and 9–32% for total phosphorus input, with substantial changes in the Bay of Biscay and Iberian Coast and in the Mediterranean region, especially by abating point sources (Tables S5.1 and S5.2 in SuppMat S5).

#### 3.2.2 Changes in nutrient loads to the sea

**Fig. 3:**
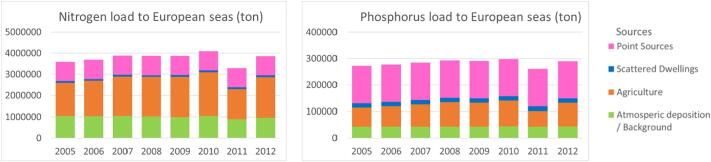
Total nitrogen (left) and phosphorus (right) annual load to European seas estimated by the GREEN model (ton/y) for the period 2005–2012. Colours indicate the relative contribution of major sources: point sources, scattered dwellings, agriculture and background (P) or atmospheric deposition (N). (For interpretation of the references to colour in this figure legend, the reader is referred to the web version of this article.)

For the period 2005–2012, the model estimated that the total nitrogen load to European seas was 3.3–4.1 TgN/y (3.9 TgN/y in 2012) and the total phosphorus load was 0.26–0.30 TgP/y (0.29 TgP/y in 2012) (Fig. 3). Considering the most recent year 2012, which was an average year for precipitation, the scenarios BAU, NUTR and MTFR bring about a reduction of nitrogen load of 2%, 6% and 14%, respectively, and a reduction of phosphorus load of 3%, 8%, 20%, respectively. The changes are related mainly to a reduction of agricultural sources for nitrogen and to an improvement of wastewater discharges for phosphorus.

**Fig. 4:**
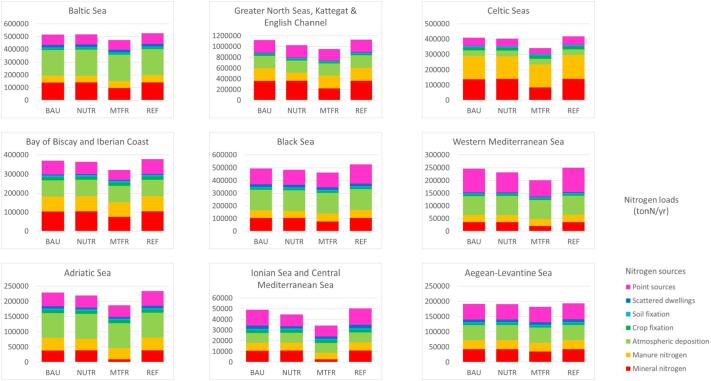
Total nitrogen load to different regional seas estimated by the GREEN model (ton/y) for the reference (REF) and the BAU, NUTR and MTFR scenarios (Table 1). Colours indicate the relative contribution of major sources: mineral nitrogen, manure nitrogen, atmospheric deposition, crop fixation, soil fixation, scattered dwellings and point sources. (For interpretation of the references to colour in this figure legend, the reader is referred to the web version of this article.)

**Fig. 5:**
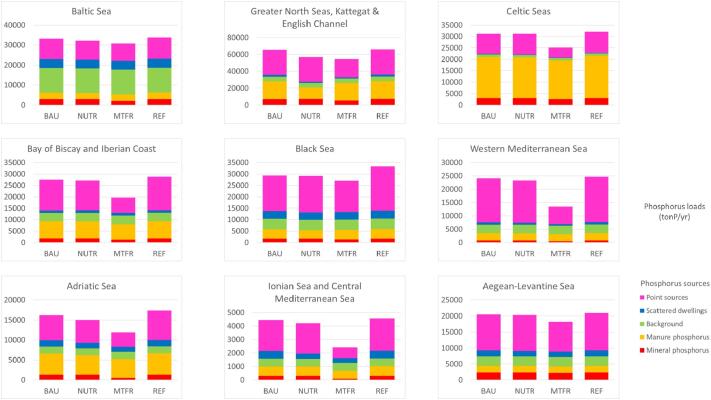
Total phosphorus load to different regional seas estimated by the GREEN model (ton/y) for the reference (REF) and the BAU, NUTR and MTFR scenarios (Table 1). Colours indicate the relative contribution of major sources: mineral phosphorus, manure phosphorus, background, scattered dwellings and point sources. (For interpretation of the references to colour in this figure legend, the reader is referred to the web version of this article.)

The scenarios produce different effects regionally (Fig. 4, Fig. 5, tables provided in SuppMat S6). The BAU scenario involves quite limited reductions for both nitrogen and phosphorus load (1%-3%) in almost all regional seas except in the Baltic Sea, where improvements are related to investments in upgrading waste water treatments, and in the Adriatic Sea (mainly for phosphorus abatement). The NUTR scenario benefits especially the Greater North Sea (-9% N load, −14% P load) for reduction of nutrients from agricultural sources, and the Central and Western Mediterranean (up to −11% for N load and −13% for P load) and the Black Sea (−6% N load, −12% P load) for improvements in wastewater treatments and agricultural sources. The highest decline of nutrient loads to European seas are obtained by the MTFR scenario, with reductions of nitrogen ranging from around 10% in the Baltic and Danube regions, to about 15% in the Greater North Sea, Celtic sea, Bay of Biscay and Iberian Coast, and 20% and more in the Central and Western Mediterranean. These improvements are related to both upgrading of wastewater treatment plants and reduction of mineral fertiliser application in the Mediterranean and Balkan regions, and mainly due to a reduction of mineral fertilisers in the Great North and Baltic regions. Concerning phosphorus, the MTFR foresaw a decrease of 9% in the Baltic Sea, around 20% in the Greater North Sea, Celtic Sea and Black Sea, and a drop of 45–47% in the Central and Western Mediterranean. Also, in this case changes are associated to reduction in the use of mineral fertiliser (in the Mediterranean and Baltic region) and to the abatement of point sources (in the Mediterranean and Bay of Biscay and Iberian Coast). Model results for different European regional seas and river basins can be explored in the online viewers associated to the data (Grizzetti et al., 2020).

#### 3.2.3 Changes in nutrient concentration and N:P ratio

The nutrient load and concentration in the scenarios depend on the combined effects of measures to combat water scarcity and actions to curb nutrient pollution. The measures implemented for water saving generally slightly increase the water flow in rivers (dilution effect for nutrients), especially in the Mediterranean region and in some irrigated basins in Western Europe, while in Eastern Europe future investments in water use are foreseen to produce a small decrease of the water flow (concentration effect for nutrients).

Mean concentrations of both nitrogen and phosphorus decrease under the three scenarios. On average, in inland waters the reduction is higher for phosphorus (up to −18% in MTFR) than for nitrogen (up to −11% in MTFR). The measures affect mainly polluted areas, with decreases observed especially in streams with concentrations above the 75th percentile. The decline in concentration is more prominent at the outlets to the sea than in inland waters, with small variation under the BAU scenario and changes of about −10% and −20% in the NUTR and the MTFR scenario respectively (SuppMat S7).

The ratio between nitrogen and phosphorus (N:P) in inland waters varies greatly across Europe, according to local conditions and nutrient sources. The effect of the scenarios on the respective proportion of the nutrient can be very different. At the sea outlets, the BAU does not substantially alter the N:P ratio, as by construction the scenario involves the same reduction of nitrogen and phosphorus in agriculture field and limited improvements in wastewater treatment plants. Conversely, overall the N:P ratio increases under the NUTR scenario and decreases under the MTFR scenario. This is related to the fact that the N:P ratio in the manure is different from that of the mineral fertilisation, with the NUTR scenario limiting manure and the MTFR tackling mineral fertilisation (SuppMat S7).

### 3.3 Link to the ecological status of water bodies

The changes in nutrient concentration and N:P ratio have implications on the ecological condition and biological components of the aquatic ecosystems. The average concentration of nitrogen and phosphorus estimated by the model GREEN for the WISE water bodies for the different class of ecological status is shown in Table 2. Considering only WISE water bodies that are currently reported in good ecological status, for year 2012 (baseline) the model estimates an average concentration of 1.13 mgN/l and 0.08 mgP/l for lakes, 2.52mgN/l and 0.16 mgP/l for rivers, and 3.88 mgN/l and 0.44 mgP/l for transitional waters. Class average nutrient concentration increases for water bodies from high to more degraded status. It also takes high values in water bodies for which the ecological status is not reported.

**Table 2:** Concentration estimated by the model GREEN per water body type and class of ecological status. Values refer to the baseline scenario (REF) in year 2012. Concentrations are weighted by the fraction of segment length or polygon area of the WISE water bodies falling in each catchment of the GREEN spatial data model. The values discussed in the text are reported in bold.

				**Mean nitrogen concentration (mgN/l)**		**Mean phosphorus concentration (mgP/l)**	
	**Class of Ecological Status**	**Total segment length (km)**	**Total polygon area (km2)**	(weighted by length)	(weighted by area)	(weighted by length)	(weighted by area)
**Lakes**							
	High	–	56,948	–	**0.80**	–	**0.09**
	Good	–	160,056	–	**1.13**	–	**0.08**
	Moderate	–	283,399	–	**1.75**	–	**0.12**
	Poor	–	111,154	–	**2.76**	–	**0.15**
	Bad	–	15,004	–	**24.31**	–	**1.28**
	Unknown	–	5392	–	**3.47**	–	**0.20**
**Rivers**							
	High	764,081	5159	**0.81**	3.17	**0.07**	0.31
	Good	2,551,093	12,467	**2.52**	3.97	**0.16**	0.38
	Moderate	5,012,752	19,176	**4.63**	4.67	**0.25**	0.42
	Poor	2,287,402	3634	**8.05**	4.41	**0.41**	0.42
	Bad	808,669	632	**10.72**	19.92	**0.60**	0.74
	Unknown	232,524	648	**4.52**	6.33	**0.26**	0.42
**Transitional waters**							
	High	8	118	–	**0.72**	–	**0.06**
	Good	74	3494	13.19	**3.88**	0.55	**0.44**
	Moderate	56	12,102	1.27	**9.16**	0.08	**1.17**
	Poor	21	7628	2.75	**14.26**	0.18	**1.17**
	Bad	–	9830	–	**12.46**	–	**1.23**
	Unknown	–	424	–	**42.03**	–	**2.77**

Based on the average concentration found by modelling for water bodies in good ecological status, the threshold of 2.0 mgN/l for nitrogen and 0.1 mgP/l for phosphorus were set to compare the potential effect of the three scenarios for reaching the policy target of good ecological status (Table 3). Overall, the predicted improvement concerns only a limited fraction of inland waters, with only an additional 1% of rivers length and 3–4% of lakes and transitional waters area being below the thresholds in the MTFR scenario. These fractions slightly increase when restricting the analysis to water bodies for which the “nutrient pollution” impact is reported in the WISE data (i.e. 2% of rivers length and 4–5% of lakes and transitional waters area).

**Table 3:** Fraction of rivers length, lakes area and transitional waters area of WISE water bodies that is below a threshold concentration of 2.0 mgN/l and 0.1 mgP/l under the reference and BAU, NUTR and MTFR scenarios (Table 1). (*) Compute on a subset of water bodies reporting 'nutrient pollution' impact in WISE data.

		**Nitrogen concentration THRESHOLD < 2.0 (mgN/l)**		**Phosphorus concentration THRESHOLD < 0.1 (mgP/l)**	
	Scenario		(*)		(*)
**Lakes**					
	REF	0.87	*0.68*	0.88	*0.70*
	BAU	0.87	*0.68*	0.88	*0.70*
	NUTR	0.88	*0.69*	0.88	*0.71*
	MTFR	0.88	*0.70*	0.89	*0.72*
**Rivers**					
	REF	0.43	*0.30*	0.46	*0.33*
	BAU	0.44	*0.31*	0.46	*0.34*
	NUTR	0.44	*0.31*	0.49	*0.37*
	MTFR	0.47	*0.34*	0.49	*0.37*
**Transitional waters**					
	REF	0.08	*0.10*	0.07	*0.10*
	BAU	0.09	*0.10*	0.07	*0.10*
	NUTR	0.09	*0.10*	0.08	*0.10*
	MTFR	0.11	*0.12*	0.11	*0.14*

## 4 Discussion

### 4.1 Modelling and scenarios novelty and limitations

Compared to previous studies and modelling assessments available in the literature at the global and continental scale (Seitzinger et al., 2005, Mayorga et al., 2010, Beusen et al., 2015, Beusen et al., 2016, Lindström et al., 2010, Donnelly et al., 2013) what is new and different in this study is the application of a straightforward modelling approach for the quantification and scenario analysis of nutrient pressures on the water environment integrating sources and sinks from the land to the sea, considering together freshwater bodies (rivers and lakes) and coastal waters, including an high spatial resolution and adopting, as much as possible, data reported by EU Member States under current EU data collection systems and regulations. Assessing nutrient pressures and the effect of policy scenarios on fresh and coastal waters at the European scale is challenging. Europe is a wide continent with a large variability of soil, climatic, hydrological and ecological features, and much diversified landscapes and agronomic production systems. Nutrient pressures on water from agriculture or point sources and remediation measures in place vary greatly according to the regional socio-economic conditions. To address the complexity of nutrient pollution in European waters, special attention was dedicated to the spatial representation of the hydrological system and coastal catchments, and to use the most updated and homogeneous data on nutrient pressures at the European scale. Both aspects are crucial for assessing policy scenarios, indeed the consistency and transparency of nutrient sources are necessary to ensure a sound representation of policy actions, and the quantification of their impacts from the single water bodies, to the river basins and marine regions is useful to support different levels of policy intervention. The high spatial resolution of model input and output (catchments of ~ 7 km^2^) afforded a detailed representation of nutrient sources and a good link to water bodies delineated by Member States under the WDF (WISE data). A specific effort was dedicated to the inclusion of coastal catchments, to improve the representation of nutrient sources in these areas, which are generally highly populated with nutrient discharges directly into the sea. Nevertheless, the model has some structural limitations, as it provides a simplified representation of nutrient processes and pathways in the aquatic environment. It does not include feedback processes such as the effect of irrigation on fertiliser input or the pollution legacy in groundwater. While nutrient retention taking place in lakes is explicitly described in the model, the effect of other watershed features affecting retention along the land-to-sea continuum, such as wetlands or drainage canals, are lumped together (Powers et al., 2015, Grizzetti et al., 2015, Hansen et al., 2018, Soana et al., 2019). In addition, the model provides annual estimations of nitrogen and phosphorus loads and their inter-annual variation but for ecological impacts seasonal distribution is also relevant.

In the assessment, nutrient concentrations result from the ratio between nutrient loads estimated by the model GREEN and water flow produced by the model LISFLOOD. In some catchments, concentrations can take high values, for examples in upstream dry areas where modelled water flow is low, or in presence of large point sources discharges, with high share of artificial water circulation, which is not well captured by the model GREEN (water abstractions and return water is taken into consideration in the computation of water flow in the model LISFLOOD).

Data availability also imposed some constraints. Observed data for model calibration were limited to the information available by the European Environment Agency (EEA), which concerned only annual nutrient concentration (without associated information on water flow) and were not homogeneously distributed across Europe. In future model calibration, statistical techniques, such as bootstrap sampling, could be used to balance the influence of heterogeneous distribution of gauging points, so that upstream and downstream stations could be proportionally represented. With regard to nutrient input, urban wastewater discharges were estimated only for year 2012 and kept constant in the simulation, disregarding the changes that have occurred between 2005 and 2012. This limits loads simulations but has no relevance for the scenarios analysis. At the time of the analysis the most recent information on the European land cover were referring to 2012 (i.e. Corine Land Cover 2012); nutrient input time series from the CAPRI model were available until 2013; most recent data reported by EU Member States on nitrogen and phosphorus discharges from wastewater treatment plants were referring to the period 2010–2012 (Vigiak et al. 2020); and water quality data of all Europe for model calibration published by EEA were available until 2012 (EEA, 2016). However, situation in 2012 could still represent the current condition as in the last decade there were not significant changes at the EU scale in the soil gross nutrient balance and indicators of nutrient concentration in rivers (EEA, 2020, Maes et al., 2020).

With regard to the analysis the novelty lies mainly in the policy dimension of the scenarios, which are conceived to represent the current planned investments (BAU) in the EU, the ambitions of EU policy in place (NUTR) and the nutrient reduction achievable by possible technological improvements (MTFR). Certainly, the scenarios are far from perfect, as the analysis has also shown the difficulty in translating measures foreseen in the last WFD RBMPs into quantitative values. All the scenarios considered nutrient reductions in both point and diffuse agricultural sources. The BAU scenario envisaged the application of measures limiting nutrient losses to water according to the current investments under the Urban Waste Water Treatment Directive (Art.17) and Rural Development Programmes (priority 4b). An average 10% reduction of the nitrogen and phosphorus entering the water system was considered in the modelling to represent the implementation of measures to reduce nutrient losses to waters based on the study of (Sarteel et al., 2016), but more regional variability is possible. It is important to note that in the BAU scenario the effect of additional measures in place under Action Programmes of the Nitrates Directive or other schemes could not be taken into consideration for the lack of consistent and quantitative information across Europe. More knowledge in this regard would benefit future assessments. The NUTR scenario focused on the reduction of manure in Nitrates Vulnerable Zones, independently from the presence of Derogations. Information on the manure was available at the NUTS2 level from the model CAPRI and was then spatialized using the Corine Land Cover map (as explained in the Section 2.3), but no information was available on the way manure is treated and stored, which can influence the impact on water pollution. The MTFR scenario minimised the use of mineral fertilisers, recycling almost all manure produced as fertiliser, but not reducing the current livestock production. The optimisation was computed using nitrogen balance at the country scale, as regional values were not available, but this might not be fully representative for countries such as Italy that have a strong gradient in fertiliser inputs across the country.

### 4.2 Nutrients loads to the European seas

According to the estimations of the BAU scenario, the current investments by the EU Member States for reducing point and diffuse nutrient pollution might result in little improvements of water quality (only 2–3% reduction of the nutrient load to the seas). There is still a potential for nutrient recovery in wastewaters that could be tapped into, especially in the Danube and the Adriatic regions. Also, in many EU countries measures to reduce nutrient losses to water could be extended, as they currently cover only a small fraction of the agricultural land (according to the budget allocation under priority 4b in Rural Development Programmes).

The NUTR and MTFR scenarios represent the possible effects of more ambitious investments. Concerning wastewater, significant nutrient reductions could be obtained in the Atlantic coasts and in the Mediterranean and Danube regions, with improvements possible also beyond the implementation of the UWWTD. However, a significant reduction of nutrient loads to the seas would only be possible with important cuts to mineral fertiliser applications, if livestock production remains unchanged (MTFR scenario), or through a lower production or a different management of manure (NUTR scenario). This could be achieved applying optimal fertilisation techniques across all Europe, adopting a better synergy between crop and livestock production and/or reducing the livestock intensity. Importantly, in the three scenarios analysed in this study, the reduction of nutrient from point sources corresponds to progressive upgrading of wastewater treatment plants, while the decrease from agricultural sources corresponds to three different strategies that could be combined, yielding to a larger nutrient decline. In addition, measures to reduce nitrogen input from atmospheric deposition where not considered, neither the model includes a feedback mechanism to simulate the positive effect of agricultural measures on limiting nitrogen emission/deposition from the atmosphere.

### 4.3 Eutrophication and policy targets

Many European seas and coastal areas suffer from problems of hypoxia and eutrophication related to high nutrient loads from the draining basins (Diaz and Rosenberg, 2008, Ménesguen and Lacroix, 2018), with large areas affected in the Baltic Sea (Meier et al., 2019, Meier et al., 2018, Skogen et al., 2014), Greater Northern Sea (Garnier et al., 2019, Lancelot et al., 2011, Passy et al., 2016, Thieu et al., 2010), Mediterranean Sea (Colella et al., 2016, Cozzi et al., 2018, Cozzi and Giani, 2011, Karydis and Kitsiou, 2012, Macias et al., 2018), and Black Sea (Kudryavtseva et al., 2019, Lancelot et al., 2002). Eutrophication is caused by excess of nitrogen and phosphorus over silica (Billen and Garnier, 2007, Howarth et al., 2011). Generally, nitrogen controls the eutrophication in coastal marine ecosystems (Howarth and Marino, 2006), while phosphorus is considered the limiting nutrient in freshwaters (Elser et al., 2007). The impact of riverine nutrient inputs on eutrophication depends on the receiving waters. In coastal marine ecosystems shelf orography, morphology, water circulation, turbidity, light and salinity, all influence the eutrophication process (Carstensen et al., 2019, Viaroli et al., 2015). For example, the marine region under the freshwater influence is larger for the Po and the Danube than for the Ebro and Rhone rivers because of the shelf orography. These rivers are characterized by an excess of dissolved inorganic nitrogen and silica over phosphate, which is sometimes compensated by the bioavailability of organic phosphorus (Cozzi et al., 2018). Anthropogenic activities have increased the amount of nutrients delivered to the aquatic environment but at the same time have also altered their balance. Similarly, measures to reduce nutrient pollution can create or exacerbate nutrient imbalance in the receiving coastal waters (Howarth and Marino, 2006). For instance, in south-eastern Europe the potential risk for coastal ecosystems eutrophication in relation to nitrogen and phosphorus has changed between the 1990 s and the 2000 s (Romero et al., 2013). For all these reasons, an important aspect to consider in the scenarios is the expected changes of the N:P ratio in the nutrient load at the sea. Overall, the scenarios indicate a higher abatement of phosphorus loads than nitrogen loads for the whole area of study (for example −20% P and −14% N in the MTFR scenario), but the impacts are very site specific and should be analysed case by case. The present analysis does not search to assess frequency or occurrences of eutrophication events (also given the limitation of an annual model), rather a potential nutrient imbalance that can favour eutrophication occurrence. Annual loads, such as those estimated in this study, can be used to inform coastal eutrophication, as shown in (Garnier et al., 2020).

Concerning the potential change in the ecological status of rivers, lakes and transitional waters the analysis suggests that an ambitious scenario (such as the MTFR) would only slightly increase the fraction of river length and water body area in good ecological status, with better effects in regions under high nutrient pressures. However, it has to be noted that the thresholds chosen for the scenarios comparison were very conservative, and were closer to the average concentration estimated for the ‘good ecological status’ class rather than to the boundary between good and moderate class. Also, in the case of freshwaters, the vulnerability of the ecosystem to nutrient enrichment strongly depends on the water body type. Across Europe, good-moderate total nitrogen threshold concentrations of 0.25–4.00 mgN/l (median 1.0 mgN/l) and total phosphorus threshold concentrations of 5–500 µgP/l (median 27.5 µgP/l) were reported per lakes, and good-moderate total nitrogen threshold concentrations of 0.25–35 mgN/l (median 2.5 mgN/l) and total phosphorus threshold concentrations of 8–660 µgP/l (median 100 µgP/l) were reported per rivers (Poikane et al., 2019a), indicating that rivers and lakes with nutrient concentrations higher than the thresholds adopted in the present analysis could also reach good ecological status. In addition, the scenarios results clearly show that the reduction in nutrient concentration mainly concerns concentrations in the 75th percentile, which indicates that ecological condition might improve in many degraded water bodies even without dropping their concentration below the chosen thresholds. Interestingly, the model GREEN captured the distribution of nutrient concentration per water body type and per class of ecological status, reporting increasing average concentrations from lakes, to river and transitional waters for both nitrogen and phosphorus, which generally corresponds to the characteristics observed for these aquatic ecosystems (Poikane et al., 2019a).

The scenarios analysed in this study were conceived to test possible effects of current and future policy actions in the EU. To curb eutrophication in fresh and coastal waters specific targets for nitrogen and phosphorus should be based on the local ecosystem condition, considering the inland sources of nutrient. The results of this study could help in this sense, allowing to link basin specific riverine nutrients per source to nutrient targets of fresh and coastal waters established under the WFD and MFSD (for nutrient targets see (Poikane et al., 2019b, Salas Herrero et al., 2019)), contemporary checking the coherence of the freshwater and marine policy objectives.

## 5 Conclusions

This study provides a picture of the major nitrogen and phosphorus sources in European river basins and the consequent pressures on fresh and coastal waters, adopting homogeneous data reported by EU and non-EU countries through standard and/or official data reporting flows. The analysis addressed the needs of model simplicity, data transparency, high spatial resolution and hydrological consistency that allow linking the riverine nutrient sources and possible measures to the ecological targets of the current EU water policies, WFD and MFSD, also checking their coherence with other sectoral policies, such as the agricultural policy.

The results of the study show that current investments in the EU countries for limiting point and diffuse nutrient pollution might result in a mild reduction of the nutrient load to the European seas, while more ambitious measures could decrease nutrient export to the sea up to 14% for nitrogen and 20% for phosphorus. Further reductions could be possible by a combination of measures especially in the agricultural sector. Importantly, future actions could widen the imbalance between nitrogen and phosphorus in receiving waters, affecting the aquatic ecosystems. In Europe regional differences and ecosystems specificity are present and need to be taken into consideration in the analysis of pressures and impacts, as well as when setting nutrient restoration targets both for freshwater and coastal ecosystems. Moreover, the results of the scenarios stimulate a reflection about the future of European aquatic ecosystems. Indeed, they highlight that the reduction of nutrient pollution from agriculture can be substantial, but in some regions, without tackling current livestock and agricultural production and consumption, the reduction might not be sufficient for achieving the goals of EU water policy.

The results of the study offer a pragmatic support to the science-policy discussion, providing the magnitude of nutrient pollution problem, the contribution of different sources, and the consequences of measures on the imbalance of nitrogen and phosphorus in water, which plays a role in eutrophication. Finally, the scenarios analysis highlighted the advantages of adopting a nexus thinking when addressing the land-sea dynamics, with measures taken in several sectors under different policies evaluated together. The scenarios implied measures for water quantity, such as irrigation efficiency and water for energy cooling, and for water quality, including actions under the UWWTP Directive, the Nitrates Directive and the CAP. The relevance of adopting a holistic approach will be key to meet the ambitious policy goals of the new European Green Deal on Zero Pollution, Farm to Fork and Biodiversity Strategy, with the objective of protecting aquatic ecosystems, ensuring their resilience and their sustainable use.

## 6 Data

The results of this study are available at the JRC Data Catalogue and can be explored in the associated online viewers and dashboards.

**Table:** 

**Topic**	**Data**
Nitrogen and Phosphorus loads to the sea (2005–2012)	https://data.jrc.ec.europa.eu/dataset/40a7c964-d62d-4a68-810e-efe026558d4f
SCENARIOS of Nitrogen and Phosphorus loads to the sea	https://data.jrc.ec.europa.eu/dataset/2ad4a18e-d953-423d-87f7-3ffdcef9bc16
Nutrient Diffuse Sources (2005–2012) Data Explorer	https://data.jrc.ec.europa.eu/dataset/e249aac0-5914-45f3-93ea-e3651a665fe1
Nutrient Diffuse Sources (SCENARIOS) Data Explorer	https://data.jrc.ec.europa.eu/dataset/0d0c374c-1843-4a1e-9ca2-0197956c9114
Nutrient Point Sources (SCENARIOS) Data Explorer	https://data.jrc.ec.europa.eu/dataset/2f4233f9-c8fe-45b2-b4bd-d26dd75bdc0c
Nutrient Concentrations (2005–2012) Data Explorer	https://data.jrc.ec.europa.eu/dataset/4643d4d4-508b-45b7-9e54-88c26c6ec203
Nutrient Concentrations (SCENARIOS) Data Explorer	https://data.jrc.ec.europa.eu/dataset/e1f0d760-8265-4c0f-bfbf-357d67aae875

## Declaration of Competing Interest

The authors declare that they have no known competing financial interests or personal relationships that could have appeared to influence the work reported in this paper.
