# Supplementary material for: How EU policies could reduce nutrient pollution in European inland and coastal waters
Source: Glob Environ Change. 2021 Jul;69:102281. doi: 10.1016/j.gloenvcha.2021.102281 (PMC8386246; doi:10.1016/j.gloenvcha.2021.102281)
Supplement: Supplementary data 1 [file mmc1.docx]

# Supplementary Material

## S1. Model GREEN equations

For each catchment *i* in the geo-data model of GREEN the nutrient load L_i_ is estimated by the **general equation**:

L_i_ = (1-Lret_i_)*(DS_i_ *(1-Bret_i_ )+ PS_i_ +U_i_ )*(1-Rret_i_) (Equation S1)

Where:

L = Nutrient load at the catchment outlet (ton/yr)

DS = Nutrient diffuse sources in the catchment (ton/yr)

PS = Nutrient point sources in the catchment (ton/yr)

U = Nutrient load from upstream catchemnts (ton/yr)

Lret = Lake retention (fraction)

Bret = Basin retention (fraction)

Rret = River retention (fraction)

Bret_i_ = 1 - exp ( -***basinCoeff*** * Inverse of precipitation_i_) (Equation S2)

Rret_i_ = 1 - exp ( -***riverCoeff*** * River length_i_) (Equation S3)

In the Equation S2 and S3 the inverse of precipitation and the river length are scaled by maximum scaling (Frank and Todeschini, 1994).

The retention occurring in lakes (Lret) was computed according to Kronvang et al. (2004), as follows:

Lret = 1 – 1/[1+(7.3/z)*RT] (for nitrogen) (Equation S4)

Lret = 1 – 1/[1+(26/z)*RT] (for phosphorus) (Equation S5)

Where:

z = average lake depth (m),

RT = hydraulic residence time (yr).

The average lake depth and hydraulic residence time were obtained from HydroLAKES databse (<https://www.hydrosheds.org/pages/hydrolakes>, Messager et al. 2016).

In **GREEN nitrogen model**, for each catchment *i* the total nitrogen load L_i_ is estimated by the equation:

L_i_ = (1-Lret_i_) * [(MinN_i_ + ManN_i_ + FixN_i_ + SoilN_i_ + (1-FF_i_)*AtmN_i_)*(1-Bret_i_) +

0.38*FF_i_*AtmN_i_ + 0.5*SdN_i_ + PsN_i_ + U_i_] * (1-Rret_i_) (Equation S6)

Where:

MinN = Nitrogen mineral fertilisers (ton/yr)

ManN = Nitrogen in manure fertilisers (ton/yr)

FixN = Nitrogen fixation by leguminous crops and fodder (ton/yr)

SoilN = Nitrogen fixation by bacteria in soils (ton/yr)

AtmN = Nitrogen deposition from atmosphese (ton/yr)

SdN = Nitrogen input from scattered dwellings (ton/yr)

PsN = Nitrogen input from point sources (ton/yr)

U = Nitrogen load from upstream cacthments (ton/yr)

FF = Non-agricultural land cover in the catchment (fraction)

Input from scattered dwelling (SD) are estimated to be reduced by 50% before entering the river

Background losses for nitrogen are estimated as 0.38*FF*AtmN. For an atmospheric deposition of 10 kgN/ha this corresponds to a background of 3.8 kgN/ha (in line with the values reported by HELCOM, 2003).

In **GREEN phosphorus model**, for each catchment i the total phosphorus load L_i_ is estimated by the equation:

L_i_ = (1-Lret_i_) * [(MinP_i_ + ManP_i_ + (1-FF_i_)*BgP_i_)*(1-Bret_i_) +

FF_i_*BgP_i_ + 0.5*SdP_i_ + PsP_i_ + U_i_] * (1-Rret_i_) (Equation S7)

MinP = Phosphorus mineral fertilisers (ton/yr)

ManP = Phosphorus in manure fertilisers (ton/yr)

BgP = Phosphorus background losses (ton/yr)

SdP = Phosphorus input from scattered dwellings (ton/yr)

PsP = Phosphorus input from point sources (ton/yr)

U = Phosphorus load from upstream catchments (ton/yr)

FF = Non-agricultural land cover in the catchment (fraction)

Input from scattered dwelling (SD) are estimated to be reduced by 50% before entering the river.

Background losses for phosphorus are estimated at 0.15 kgP/ha (in line with the values reported by HELCOM, 2003).

**References**

Frank, I.E. and Todeschini, R., 1994. The data analysis handbook. Elsevier, Amsterdam, the Neatherlands.

Kronvang, B., Hezlar, J., Boers, P., Jansen, J. P., Behrendt, H., Anderson, T., Arheimer, B., Venohr, M., Hoffmann, C.C., 2004. Nutrient retention handbook. Software manual for EUROHARP NUTRET and scientific review on nutrient retention, no. 9-2004 in EUROHARP report, Oslo, <http://www.euroharp.org>.

HELCOM, Helsinki Commission, 2003. Fourth Baltic Sea Load Compilation. Balt. Sea Environ. Proc. No. 93. 189pp.

Messager, M.L., Lehner, B., Grill, G., Nedeva, I., Schmitt, O., 2016: Estimating the volume and age of water stored in global lakes using a geo-statistical approach. Nature Communications: 13603. doi: 10.1038/ncomms13603

## S2. Number of observations for model calibration

Table S2.1 Number and average area of catchments in the geo-spatial model of GREEN

| Number of catchments | Total Area (km2) | Average catchment area (km2) |
| --- | --- | --- |
| 950472 | 6327575 | 6.66 |

Table S2.2 Number of observed nitrogen and phosphorus concentration per year available for the calibration of the model GREEN.

| Year | Number observations Total Nitrogen | Number observations Total Phosphorus |
| --- | --- | --- |
| 2008 | 2492 | 2837 |
| 2009 | 2018 | 2186 |
| 2010 | 1564 | 2894 |
| 2011 | 1578 | 2941 |
| 2012 | 1683 | 3032 |

Table S2.3 Number of observed nitrogen and phosphorus concentration per country per year available for the calibration of the model GREEN.

|  | | | **Number observations Total Nitrogen** | | | | | **Number observations Total Phosphorus** | | | | |
| --- | --- | --- | --- | --- | --- | --- | --- | --- | --- | --- | --- | --- |
| **Country** | **N. catchments** | **Area (km2)** | **2008** | **2009** | **2010** | **2011** | **2012** | **2008** | **2009** | **2010** | **2011** | **2012** |
| AD | 71 | 93 | 0 | 0 | 0 | 0 | 0 | 0 | 0 | 0 | 0 | 0 |
| AL | 2761 | 5676 | 0 | 0 | 0 | 0 | 0 | 17 | 4 | 4 | 3 | 3 |
| AT | 7354 | 16768 | 3 | 3 | 3 | 3 | 2 | 40 | 45 | 45 | 39 | 39 |
| BA | 3340 | 10222 | 2 | 11 | 9 | 11 | 14 | 10 | 11 | 9 | 11 | 14 |
| BE | 481 | 6149 | 39 | 40 | 39 | 66 | 66 | 39 | 40 | 39 | 66 | 66 |
| BG | 3308 | 22219 | 32 | 29 | 36 | 52 | 54 | 2 | 31 | 32 | 52 | 55 |
| BY | 1956 | 17624 | 0 | 0 | 0 | 0 | 0 | 0 | 0 | 0 | 0 | 0 |
| CH | 3979 | 8286 | 6 | 0 | 6 | 48 | 48 | 8 | 7 | 8 | 88 | 87 |
| CY | 179 | 1850 | 12 | 15 | 0 | 0 | 0 | 12 | 15 | 17 | 15 | 18 |
| CZ | 2032 | 15784 | 56 | 0 | 0 | 0 | 0 | 57 | 1 | 1 | 1 | 1 |
| DE | 7394 | 71457 | 176 | 171 | 177 | 183 | 152 | 179 | 199 | 209 | 207 | 190 |
| DK | 493 | 8631 | 19 | 19 | 19 | 18 | 18 | 19 | 19 | 19 | 18 | 18 |
| EE | 707 | 9094 | 44 | 44 | 45 | 41 | 43 | 44 | 44 | 45 | 41 | 43 |
| ES | 17119 | 99576 | 166 | 178 | 184 | 0 | 80 | 197 | 169 | 246 | 87 | 92 |
| FI | 9388 | 67878 | 93 | 87 | 85 | 84 | 85 | 93 | 88 | 86 | 85 | 85 |
| FR | 17879 | 109755 | 1000 | 862 | 1 | 2 | 2 | 1001 | 854 | 1013 | 1015 | 1015 |
| GB | 5586 | 48865 | 147 | 157 | 216 | 206 | 176 | 154 | 161 | 233 | 227 | 199 |
| GG | 3 | 14 | 0 | 0 | 0 | 0 | 0 | 0 | 0 | 0 | 0 | 0 |
| GR | 8894 | 26311 | 0 | 0 | 0 | 0 | 0 | 43 | 0 | 0 | 0 | 0 |
| HR | 2453 | 11271 | 17 | 16 | 15 | 15 | 16 | 17 | 16 | 15 | 15 | 16 |
| HU | 1123 | 18519 | 7 | 6 | 5 | 5 | 5 | 8 | 8 | 6 | 5 | 5 |
| IE | 1697 | 13962 | 0 | 0 | 12 | 12 | 15 | 0 | 0 | 20 | 17 | 25 |
| IM | 14 | 114 | 0 | 0 | 0 | 0 | 0 | 0 | 0 | 0 | 0 | 0 |
| IT | 18650 | 59997 | 350 | 89 | 416 | 462 | 430 | 484 | 90 | 500 | 553 | 537 |
| JE | 2 | 24 | 0 | 0 | 0 | 0 | 0 | 0 | 0 | 0 | 0 | 0 |
| KS | 410 | 2172 | 0 | 0 | 0 | 0 | 0 | 19 | 19 | 18 | 18 | 18 |
| LI | 8 | 34 | 0 | 0 | 0 | 0 | 0 | 2 | 2 | 2 | 2 | 0 |
| LT | 1181 | 13132 | 30 | 30 | 32 | 35 | 32 | 30 | 30 | 32 | 35 | 32 |
| LU | 63 | 514 | 0 | 0 | 0 | 3 | 3 | 3 | 3 | 3 | 3 | 3 |
| LV | 1013 | 12848 | 26 | 23 | 2 | 2 | 2 | 26 | 23 | 2 | 2 | 2 |
| MD | 225 | 2930 | 0 | 0 | 0 | 0 | 0 | 0 | 0 | 0 | 0 | 0 |
| ME | 1315 | 2857 | 0 | 0 | 0 | 0 | 0 | 0 | 0 | 0 | 0 | 0 |
| MK | 1555 | 5052 | 0 | 0 | 0 | 0 | 0 | 0 | 0 | 0 | 0 | 0 |
| MT | 5 | 63 | 0 | 0 | 0 | 0 | 0 | 0 | 0 | 0 | 0 | 0 |
| NL | 216 | 6918 | 10 | 10 | 10 | 4 | 4 | 10 | 10 | 10 | 4 | 4 |
| NO | 11763 | 64782 | 29 | 29 | 29 | 28 | 48 | 29 | 29 | 29 | 28 | 49 |
| PL | 5107 | 62372 | 23 | 21 | 5 | 81 | 170 | 24 | 20 | 5 | 81 | 168 |
| PT | 2815 | 17709 | 14 | 12 | 14 | 3 | 10 | 17 | 29 | 30 | 9 | 34 |
| RO | 6092 | 47769 | 48 | 49 | 58 | 64 | 65 | 71 | 71 | 70 | 67 | 69 |
| RS | 1971 | 15539 | 34 | 43 | 44 | 43 | 32 | 43 | 43 | 44 | 43 | 32 |
| RU | 19148 | 142092 | 1 | 1 | 1 | 1 | 2 | 1 | 1 | 1 | 1 | 2 |
| SE | 9271 | 89903 | 46 | 44 | 76 | 74 | 74 | 75 | 74 | 76 | 74 | 74 |
| SI | 1331 | 4060 | 8 | 6 | 9 | 11 | 4 | 8 | 6 | 8 | 10 | 5 |
| SK | 2009 | 9735 | 53 | 22 | 15 | 20 | 29 | 54 | 23 | 16 | 18 | 30 |
| SM | 2 | 13 | 0 | 0 | 0 | 0 | 0 | 0 | 0 | 0 | 0 | 0 |
| TR | 6563 | 102218 | 0 | 0 | 0 | 0 | 0 | 0 | 0 | 0 | 0 | 0 |
| UA | 948 | 9492 | 1 | 1 | 1 | 1 | 2 | 1 | 1 | 1 | 1 | 2 |

## S3. Model calibration

Figure S3.1 GREEN model calibration: Nitrogen loads


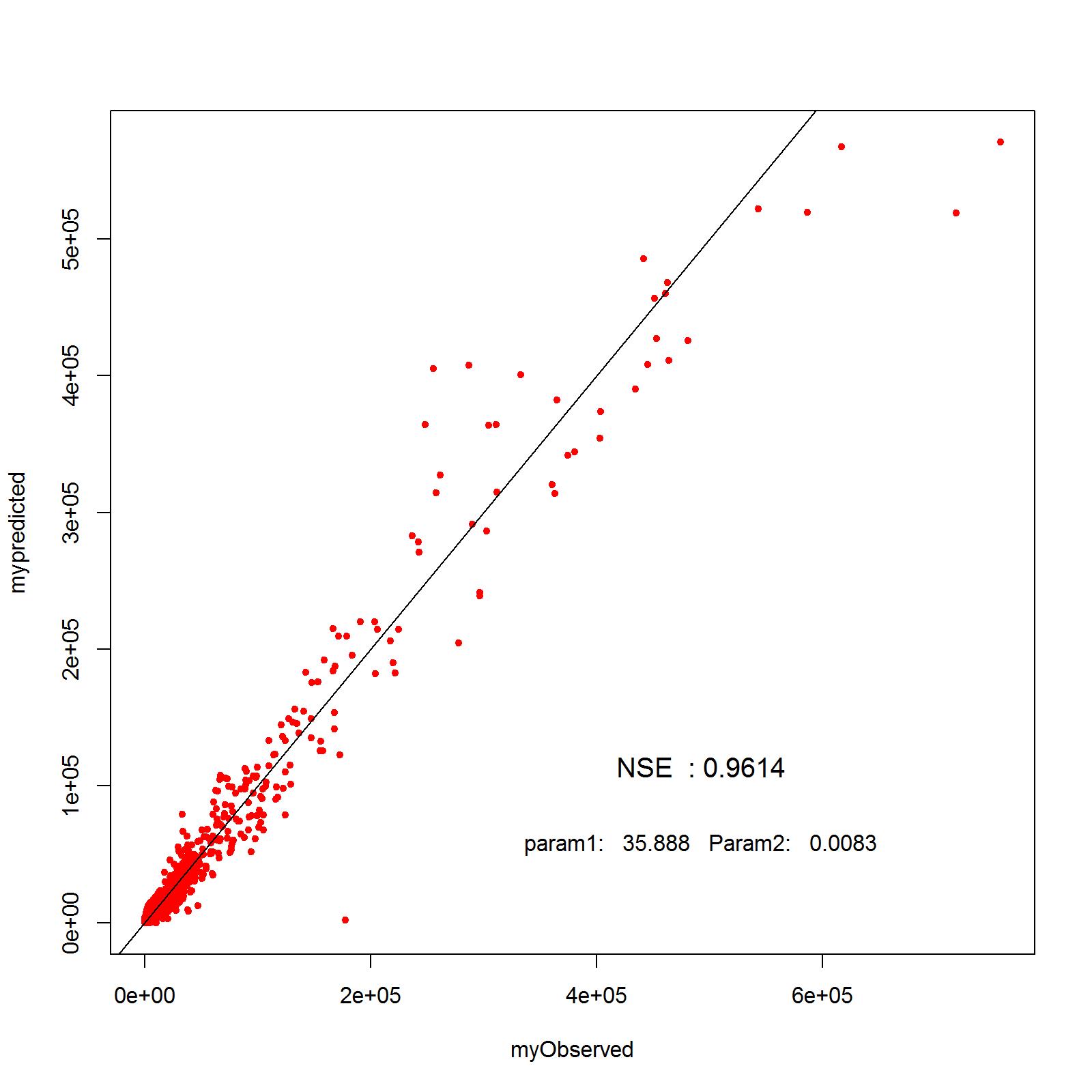


| **Period of calibration** | **Number iterations** | **basinCoeff interval** | **riverCoeff interval** | **sdCoeff** | **basinCoeff** | **riverCoeff** | **NSE (noLOG)** |
| --- | --- | --- | --- | --- | --- | --- | --- |
| 2008-2012 | 500 | 30-50 | 0.005-0.1 | 0.666667 | 35.8880 | 0.0083 | 0.9614 |

Figure S3.2 GREEN model calibration: Phosphorus loads

**
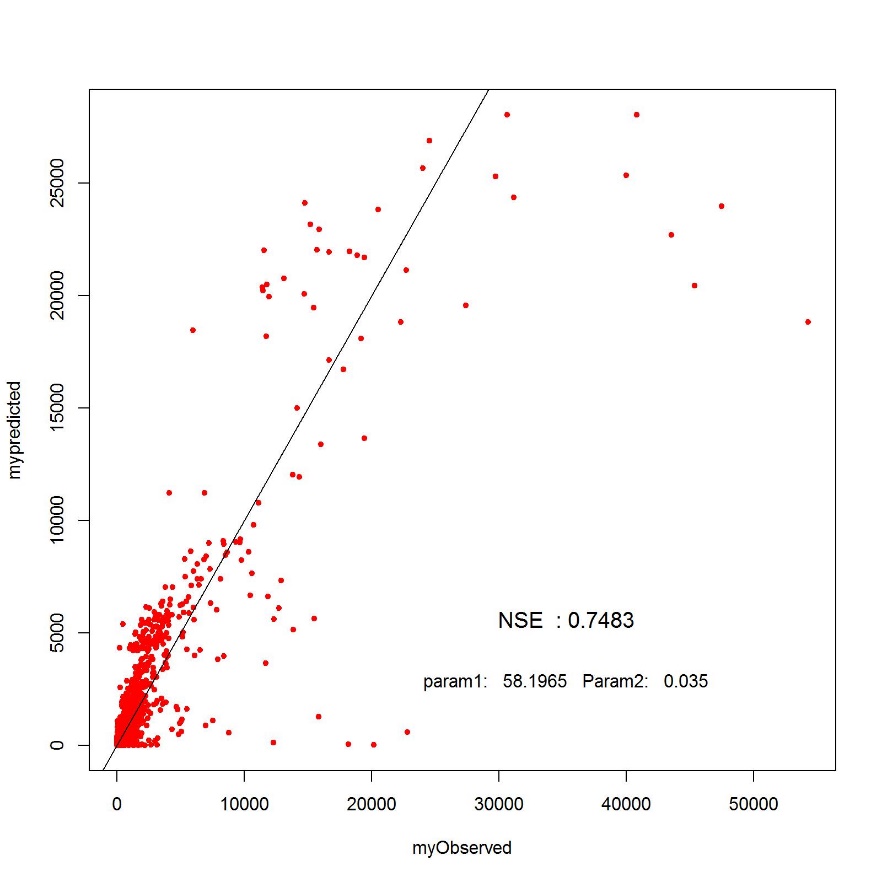
**

| **Period of calibration** | **Number iterations** | **basinCoeff interval** | **riverCoeff interval** | **sdCoeff** | **basinCoeff** | **riverCoeff** | **NSE (noLOG)** |
| --- | --- | --- | --- | --- | --- | --- | --- |
| 2008-2012 | 500 | 40-75 | 0.005-0.1 | 0.714286 | 58.1965 | 0.0350 | 0.7483 |

## S4. Model validation

Figure S4.1 The maps of the 50 European rivers (with the largest nitrogen load) where freshwater runoff and nutrient loads entering the European seas from the reference simulation of the model GREEN were compared with a variety of observed data from independent sources.  The colors refer to the different size of the catchment and are only chosen to allow distinguishing small (yellow), medium (orange) and large size (red) catchments.


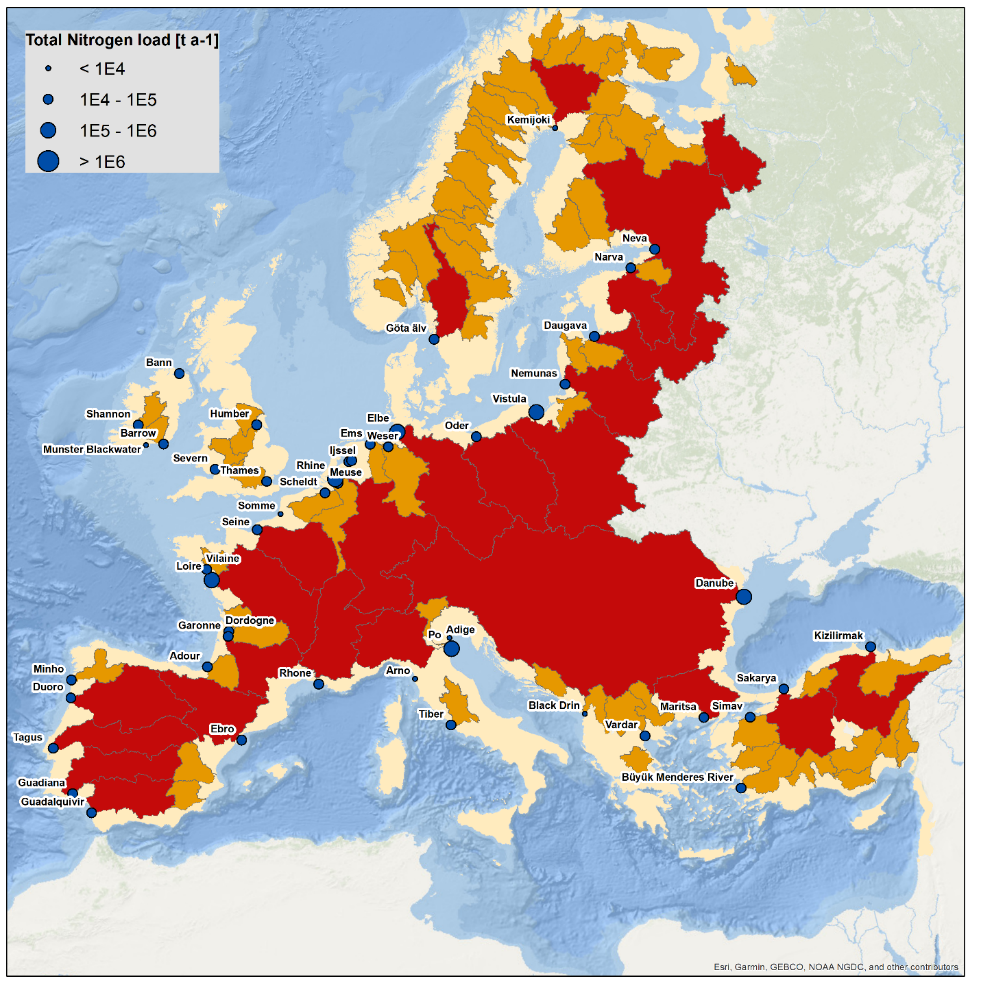


Table S4.1 Sources of data of water runoff and nutrient loads used to compared the results of the model GREEN.

| **Reference** | **Web link** |
| --- | --- |
| Global Runoff Data Centre (GRDC) | <https://www.bafg.de/GRDC/EN/01_GRDC/grdc_node.html> |
| Global Nutrient Export from WaterSheds 2 (NEWS 2; (Mayorga et al., 2010)) | <https://doi.org/10.1016/j.envsoft.2010.01.007> |
| European Environment Agency (EEA) | <https://www.eea.europa.eu/data-and-maps/data/waterbase-rivers-10> |
| Helsinki Commission (HELCOM; HELCOM (2015): | <https://helcom.fi/wp-content/uploads/2019/08/BSEP145_Lowres.pdf> |
| HELCOM (2018) | <https://helcom.fi/wp-content/uploads/2019/12/BSEP163.pdf> |
| HELCOM Map and Data Service | <http://maps.helcom.fi/website/mapservice/> |
| OSPAR Data and Information Management System | <https://odims.ospar.org/> |
| UK National River Flow Archive | <https://nrfa.ceh.ac.uk/data/search> |
| Hydro-Data provided by the Irish Office of Public Works | [http://waterlevel.ie/hydro-data/list.html#](http://waterlevel.ie/hydro-data/list.html) |
| OSPAR Intersessional Correspondence Group on Eutrophication Modelling (ICG-EMO; provided by Sonja van Leeuwen (NIOZ, pers. comm.), extension of Lenhart et al. (2010) | <https://doi.org/10.1016/j.jmarsys.2009.12.014> |

Table S4.2. Literature sources to compare freshwater runoff and nutrient loads of selected rivers with the results of the model GREEN.

| **Reference** | **River** | **Web link** |
| --- | --- | --- |
| (Cozzi et al., 2018) | Danube, Ebro, Po, Rhone | doi:10.3390/w11010001 |
| (Friedland et al., 2019) | Oder | <https://doi.org/10.3389/fmars.2018.00521> |
| (Hartmann et al., 2011) | Rhine | <https://doi.org/10.1007/s10201-010-0322-4> |
| (Hesse and Krysanova, 2016) | Elbe | <https://doi.org/10.3390/w8020040> |
| (Howden et al., 2010) | Thames | DOI: 10.1002/hyp.7835 |
| (Karydis and Kitsiou, 2012) | Adige, Drin, Ebro, Po, Rhone, Tiber | DOI 10.1007/s10661-011-2313-2 |
| (Kauppila and Koskiaho, 2003) | Kemijoki | <https://doi.org/10.2166/nh.2003.0004> |
| (Lajaunie-Salla et al., 2018) | Garonne | <https://doi.org/10.1007/s11356-018-3035-6> |
| (Lassaletta et al., 2012) | Ebro | doi:10.5194/bg-9-57-2012 |
| (Ludwig et al., 2010) | Adige, Arno, Danube, Ebro, Po, Rhone | doi:10.1029/2009GB003594 |
| (Ménesguen et al., 2019) | Dordogne, Garonne, Loire, Seine | <https://doi.org/10.1016/j.ocemod.2018.11.002> |
| (Minaudo et al., 2015) | Loire | doi:10.5194/bg-12-2549-2015 |
| (Mockler et al., 2017) | Shannon | <http://dx.doi.org/10.1016/j.scitotenv.2017.05.186> |
| (Passy et al., 2013) | Scheldt, Seine, Somme | <http://dx.doi.org/10.1016/j.jmarsys.2013.05.005> |
| (Passy et al., 2016) | Seine | doi: 10.3354/meps11533 |
| (Petus et al., 2014) | Adour | <http://dx.doi.org/10.1016/j.csr.2013.11.011> |
| (Tockner et al., 2009) | Danube, Daugava, Duoro, Ebro, Elbe, Loire, Nemunas, Neva, Oder, Rhine, Rhone, Vistula | Tockner, Klement, Urs Uehlinger, and Christopher T. Robinson. Rivers of Europe. Academic Press, 2009. |
| (Radach and Pätsch, 2007) | Elbe, Ems, Rhine, Weser | DOI: 10.1007/BF02782968 |
| (Romero et al., 2013) | Adige, Adour, Arno, Dordogne, Duoro, Ebro, Garonne, Loire, Po, Rhone, Scheldt, Seine, Somme, Tagus, Tiber, Vilaine | DOI 10.1007/s10533-012-9778-0 |
| (Skarbøvik et al., 2014) | Black Drin | DOI:10.2298/ABS1402667S |
| (Thieu et al., 2010) | Scheldt, Seine, Somme | doi:10.1016/j.scitotenv.2009.12.031 |
| (Valsecchi et al., 2015) | Adige, Arno, Po | <http://dx.doi.org/10.1016/j.chemosphere.2014.07.044> |
| (Vybernaite-Lubiene et al., 2018) | Nemunas | doi:10.3390/w10091178 |
| (Ylöstalo et al., 2016) | Neva | <http://dx.doi.org/10.1016/j.marchem.2016.07.004> |

Figure S4.2 Comparison of reported freshwater runoff with GREEN model [m3 s-1] (derived from the model LISFLOOD), shown on a log-log-scale, color-coded are the different sources for the reported values.


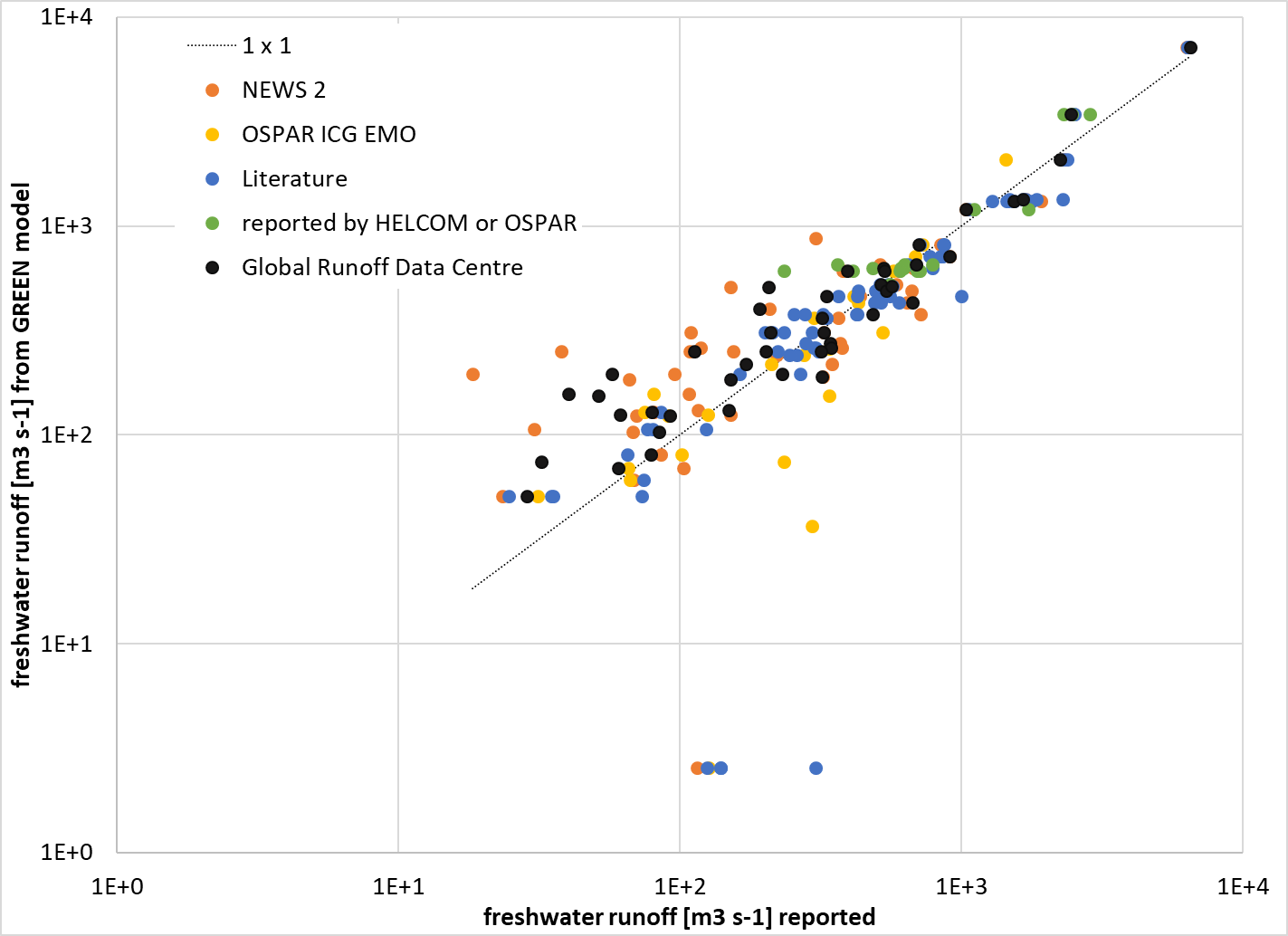


Figure S4.3 Comparison of reported annual Total Phosphorus load with GREEN model [t a-1], shown on a log-log-scale, color-coded are the different sources for the reported values.


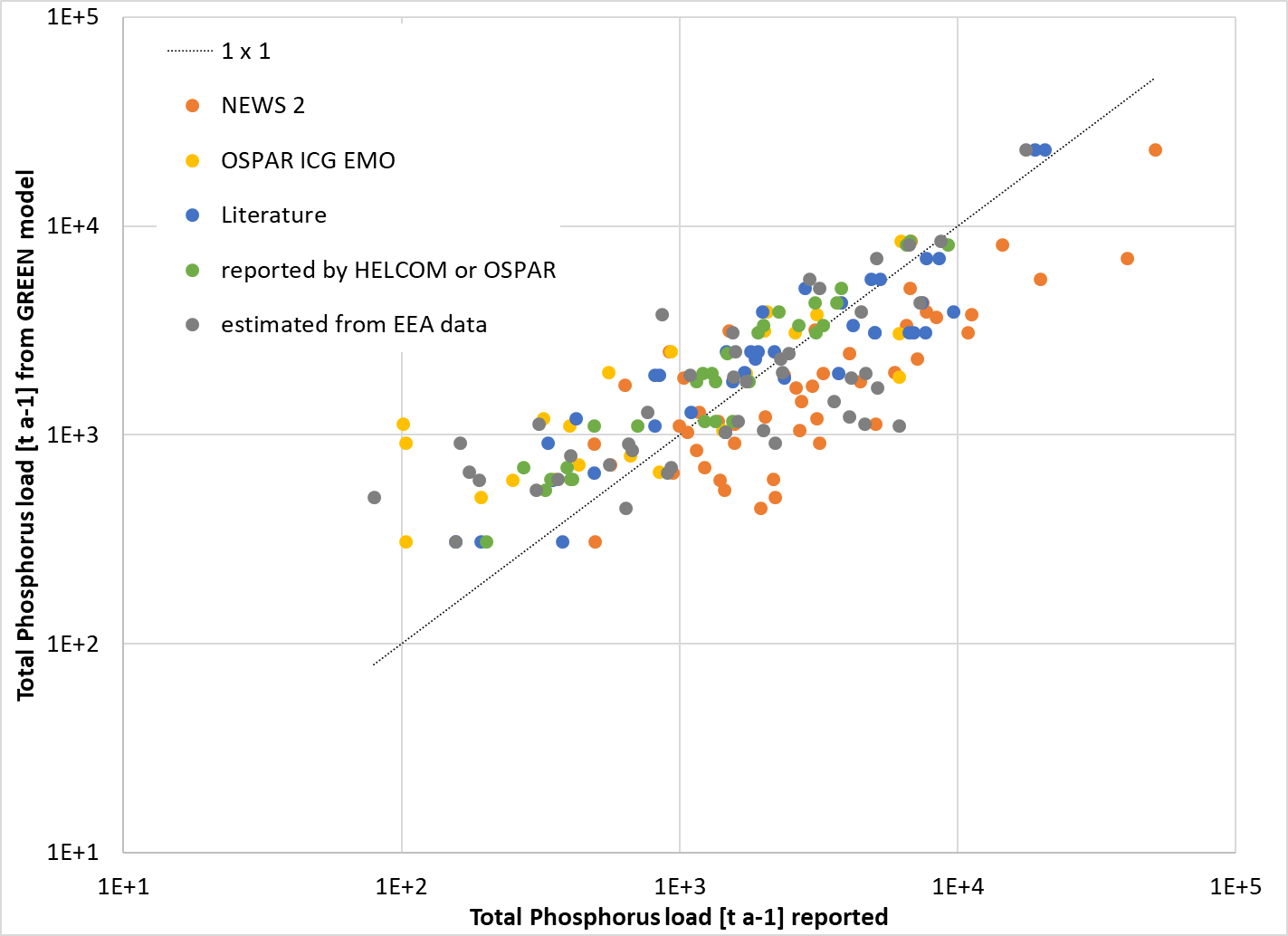


Table S4.3 Correlation coefficients (R2) and gradients of a linear fit (a) for freshwater runoff and nutrient loads between GREEN model and different data sources (listed in Table S4.1 and Table S4.2). The correlation was computed for the whole data set as well as for the different data sources separately. The linear fit between reported and modeled freshwater runoff and nutrient loads was computed, assuming the intersect at 0. *Data sources from the literature per river basin are provided in Table S4.2.

|  | Freshwater Runoff | | Total Nitrogen load | | Total Phosphorus load | |
| --- | --- | --- | --- | --- | --- | --- |
|  | R2 | a | R2 | a | R2 | a |
| All data | 0.96 | 1.079 | 0.90 | 0.961 | 0.49 | 0.565 |
| All data without NEWS2 |  |  |  |  | 0.80 | 0.993 |
| NEWS2 (global database) | 0.95 | 1.079 | 0.91 | 1.084 | 0.72 | 0.371 |
| GRDC (Global Runoff Database) | 0.97 | 1.086 |  |  |  |  |
| OSPAR ICG EMO | 0.89 | 1.174 | 0.74 | 1.085 | 0.63 | 1.078 |
| Reported by HELCOM or OSPAR | 0.90 | 1.210 | 0.85 | 1.020 | 0.90 | 1.146 |
| EEA data |  |  | 0.93 | 0.969 | 0.75 | 1.006 |
| Data from the literature* | 0.96 | 1.059 | 0.94 | 0.894 | 0.84 | 0.968 |

**References**

Cozzi, S., Ibáñez, C., Lazar, L., Raimbault, P., Giani, M., 2018. Flow regime and nutrient-loading trends from the largest South European watersheds: Implications for the productivity of mediterranean and Black Sea’s Coastal Areas. Water (Switzerland) 11. https://doi.org/10.3390/w11010001

Friedland, R., Schernewski, G., Gräwe, U., Greipsland, I., Palazzo, D., Pastuszak, M., 2019. Managing eutrophication in the Szczecin (Oder) lagoon-development, present state and future perspectives. Front. Mar. Sci. 5. https://doi.org/10.3389/fmars.2018.00521

Hartmann, J., Levy, J., Kempe, S., 2011. Increasing dissolved silica trends in the Rhine River: An effect of recovery from high P loads? Limnology 12, 63–73. https://doi.org/10.1007/s10201-010-0322-4

Hesse, C., Krysanova, V., 2016. Modeling climate and management change impacts on water quality and in-stream processes in the Elbe river basin. Water (Switzerland) 8. https://doi.org/10.3390/w8020040

Howden, N.J.K., Burt, T.P., Worrall, F., Whelan, M.J., Bieroza, M., 2010. Nitrate concentrations and fluxes in the River Thames over 140 years (1868-2008): Are increases irreversible? Hydrol. Process. 24, 2657–2662. https://doi.org/10.1002/hyp.7835

Karydis, M., Kitsiou, D., 2012. Eutrophication and environmental policy in the Mediterranean Sea: a review. Environ. Monit. Assess. 184, 4931–4984. https://doi.org/10.1007/s10661-011-2313-2

Kauppila, P., Koskiaho, J., 2003. Evaluation of Annual Loads of Nutrients and Suspended Solids in Baltic Rivers. Hydrol. Res. 34, 203–220. https://doi.org/10.2166/nh.2003.0004

Lajaunie-Salla, K., Sottolichio, A., Schmidt, S., Litrico, X., Binet, G., Abril, G., 2018. Future intensification of summer hypoxia in the tidal Garonne River (SW France) simulated by a coupled hydro sedimentary-biogeochemical model. Environ. Sci. Pollut. Res. 25, 31957–31970. https://doi.org/10.1007/s11356-018-3035-6

Lassaletta, L., Romero, E., Billen, G., Garnier, J., García-Gómez, H., Rovira, J.V., 2012. Spatialized N budgets in a large agricultural Mediterranean watershed: High loading and low transfer. Biogeosciences 9, 57–70. https://doi.org/10.5194/bg-9-57-2012

Ludwig, W., Bouwman, A.F., Dumont, E., Lespinas, F., 2010. Water and nutrient fluxes from major Mediterranean and Black Sea rivers: Past and future trends and their implications for the basin-scale budgets. Global Biogeochem. Cycles 24. https://doi.org/10.1029/2009GB003594

Mayorga, E., Seitzinger, S.P., Harrison, J.A., Dumont, E., Beusen, A.H.W., Bouwman, A.F., Fekete, B.M., Kroeze, C., Van Drecht, G., 2010. Global Nutrient Export from WaterSheds 2 (NEWS 2): Model development and implementation. Environ. Model. Softw. 25, 837–853. https://doi.org/10.1016/j.envsoft.2010.01.007

Ménesguen, A., Dussauze, M., Dumas, F., Thouvenin, B., Garnier, V., Lecornu, F., Répécaud, M., 2019. Ecological model of the Bay of Biscay and English Channel shelf for environmental status assessment part 1: Nutrients, phytoplankton and oxygen. Ocean Model. 133, 56–78. https://doi.org/10.1016/J.OCEMOD.2018.11.002

Minaudo, C., Meybeck, M., Moatar, F., Gassama, N., Curie, F., 2015. Eutrophication mitigation in rivers: 30 years of trends in spatial and seasonal patterns of biogeochemistry of the Loire River (1980–2012). Biogeosciences 12, 2549–2563. https://doi.org/10.5194/bg-12-2549-2015

Mockler, E.M., Deakin, J., Archbold, M., Gill, L., Daly, D., Bruen, M., 2017. Sources of nitrogen and phosphorus emissions to Irish rivers and coastal waters: Estimates from a nutrient load apportionment framework. Sci. Total Environ. 601–602, 326–339. https://doi.org/https://doi.org/10.1016/j.scitotenv.2017.05.186

Passy, P., Gypens, N., Billen, G., Garnier, J., Thieu, V., Rousseau, V., Callens, J., Parent, J.-Y., Lancelot, C., 2013. A model reconstruction of riverine nutrient fluxes and eutrophication in the Belgian Coastal Zone since 1984. J. Mar. Syst. 128, 106–122. https://doi.org/10.1016/j.jmarsys.2013.05.005

Passy, P., Le Gendre, R., Garnier, J., Cugier, P., Callens, J., Paris, F., Billen, G., Riou, P., Romero, E., 2016. Eutrophication modelling chain for improved management strategies to prevent algal blooms in the Bay of Seine. Mar. Ecol. Prog. Ser. 543, 107–125. https://doi.org/10.3354/meps11533

Petus, C., Marieu, V., Novoa, S., Chust, G., Bruneau, N., Froidefond, J.-M., 2014. Monitoring spatio-temporal variability of the Adour River turbid plume (Bay of Biscay, France) with MODIS 250-m imagery. Cont. Shelf Res. 74, 35–49. https://doi.org/https://doi.org/10.1016/j.csr.2013.11.011

Radach, G., Pätsch, J., 2007. Variability of continental riverine freshwater and nutrient inputs into the North Sea for the years 1977–2000 and its consequences for the assessment of eutrophication. Estuaries and Coasts 30, 66–81. https://doi.org/10.1007/BF02782968

Romero, E., Garnier, J., Lassaletta, L., Billen, G., Le Gendre, R., Riou, P., Cugier, P., 2013. Large-scale patterns of river inputs in southwestern Europe: seasonal and interannual variations and potential eutrophication effects at the coastal zone. Biogeochemistry 113, 481–505. https://doi.org/10.1007/s10533-012-9778-0

Skarbøvik, E., Perović, A., Shumka, S., Nagothu, U.S., 2014. Nutrient inputs, trophic status and water management challenges in the transboundary Lake Skadar/Shkodra, Western Balkans. Arch. Biol. Sci. 66, 667–681.

Thieu, V., Garnier, J., Billen, G., 2010. Assessing the effect of nutrient mitigation measures in the watersheds of the Southern Bight of the North Sea. Sci. Total Environ. 408, 1245–1255. https://doi.org/https://doi.org/10.1016/j.scitotenv.2009.12.031

Tockner, K., Uehlinger, U., Robinson, C.T., 2009. Rivers of Europe. Academic Press.

Valsecchi, S., Rusconi, M., Mazzoni, M., Viviano, G., Pagnotta, R., Zaghi, C., Serrini, G., Polesello, S., 2015. Occurrence and sources of perfluoroalkyl acids in Italian river basins. Chemosphere 129, 126–134. https://doi.org/https://doi.org/10.1016/j.chemosphere.2014.07.044

Vybernaite-Lubiene, I., Zilius, M., Saltyte-Vaisiauske, L., Bartoli, M., 2018. Recent Trends (2012–2016) of N, Si, and P Export from the Nemunas River Watershed: Loads, Unbalanced Stoichiometry, and Threats for Downstream Aquatic Ecosystems. Water . https://doi.org/10.3390/w10091178

Ylöstalo, P., Seppälä, J., Kaitala, S., Maunula, P., Simis, S., 2016. Loadings of dissolved organic matter and nutrients from the Neva River into the Gulf of Finland – Biogeochemical composition and spatial distribution within the salinity gradient. Mar. Chem. 186, 58–71. https://doi.org/https://doi.org/10.1016/j.marchem.2016.07.004

## S5. Nutrient inland input under different scenarios

Table S5.1 Nitrogen inland input per regional seas (per source and scenario).

| \| **Nitrogen inland input per regional seas (per source and scenario)** \| \| \| \| \| \| \| \| \| \| \| --- \| --- \| --- \| --- \| --- \| --- \| --- \| --- \| --- \| --- \| \|  \|  \| **Atmospheric deposition** \| **Scattered dwellings** \| **Point sources** \|  \| **Agriculture (Min, Man, BNF)** \|  \| **Total Input** \|  \| \|  \| Scenario \| (ton/y) \| (ton/y) \| (ton/y) \| % of change \| (ton/y) \| % of change \| (ton/y) \| % of change \| \| Baltic \|  \|  \|  \|  \|  \|  \|  \|  \|  \| \|  \| REF \| 989390 \| 33773 \| 90578 \|  \| 3365835 \|  \| 4479576 \|  \| \|  \| BAU \| 971834 \| 33730 \| 88588 \| -2 \| 3312010 \| -2 \| 4406162 \| -2 \| \|  \| NUTR \| 989390 \| 32746 \| 86179 \| -5 \| 3298474 \| -2 \| 4406788 \| -2 \| \|  \| MTFR \| 989390 \| 32746 \| 83618 \| -8 \| 2618555 \| -22 \| 3724308 \| -17 \| \| Greater North Sea \| \| \|  \|  \|  \|  \|  \|  \|  \| \|  \| REF \| 1120137 \| 15805 \| 245245 \|  \| 6284123 \|  \| 7665310 \|  \| \|  \| BAU \| 1108643 \| 15805 \| 245200 \| 0 \| 6217190 \| -1 \| 7586839 \| -1 \| \|  \| NUTR \| 1120137 \| 13161 \| 235980 \| -4 \| 5422629 \| -14 \| 6791907 \| -11 \| \|  \| MTFR \| 1120137 \| 13161 \| 214615 \| -12 \| 4857025 \| -23 \| 6204937 \| -19 \| \| Celtic Sea \| \|  \|  \|  \|  \|  \|  \|  \|  \| \|  \| REF \| 151298 \| 1755 \| 55090 \|  \| 1635782 \|  \| 1843925 \|  \| \|  \| BAU \| 148476 \| 1755 \| 52685 \| -4 \| 1604992 \| -2 \| 1807908 \| -2 \| \|  \| NUTR \| 151298 \| 1253 \| 48805 \| -11 \| 1592667 \| -3 \| 1794024 \| -3 \| \|  \| MTFR \| 151298 \| 1253 \| 37532 \| -32 \| 1367870 \| -16 \| 1557953 \| -16 \| \| Bay of Biscay and Iberian Coast \| \| \| \|  \|  \|  \|  \|  \|  \| \|  \| REF \| 444798 \| 8008 \| 79653 \|  \| 2970126 \|  \| 3502585 \|  \| \|  \| BAU \| 439683 \| 7947 \| 75373 \| -5 \| 2934104 \| -1 \| 3457107 \| -1 \| \|  \| NUTR \| 444798 \| 7908 \| 65736 \| -17 \| 2963165 \| 0 \| 3481606 \| -1 \| \|  \| MTFR \| 444798 \| 7908 \| 55760 \| -30 \| 2313345 \| -22 \| 2821811 \| -19 \| \| Black Sea \| \|  \|  \|  \|  \|  \|  \|  \|  \| \|  \| REF \| 959768 \| 38907 \| 178796 \|  \| 3820915 \|  \| 4998386 \|  \| \|  \| BAU \| 947052 \| 38405 \| 151675 \| -15 \| 3782809 \| -1 \| 4919940 \| -2 \| \|  \| NUTR \| 959768 \| 36305 \| 143400 \| -20 \| 3724705 \| -3 \| 4864178 \| -3 \| \|  \| MTFR \| 959768 \| 36305 \| 141393 \| -21 \| 3212028 \| -16 \| 4349494 \| -13 \| \| Aegean-Levantine Sea \| \| \|  \|  \|  \|  \|  \|  \|  \| \|  \| REF \| 213160 \| 14123 \| 54715 \|  \| 969524 \|  \| 1251522 \|  \| \|  \| BAU \| 211992 \| 14123 \| 52772 \| -4 \| 963854 \| -1 \| 1242740 \| -1 \| \|  \| NUTR \| 213160 \| 13011 \| 52447 \| -4 \| 967552 \| 0 \| 1246170 \| 0 \| \|  \| MTFR \| 213160 \| 13011 \| 52277 \| -4 \| 813571 \| -16 \| 1092019 \| -13 \| \| Ionian Sea and Central Med Sea \| \| \| \|  \|  \|  \|  \|  \|  \| \|  \| REF \| 40239 \| 4939 \| 15504 \|  \| 238346 \|  \| 299028 \|  \| \|  \| BAU \| 39425 \| 4933 \| 14911 \| -4 \| 233376 \| -2 \| 292645 \| -2 \| \|  \| NUTR \| 40239 \| 3229 \| 11218 \| -28 \| 235555 \| -1 \| 290241 \| -3 \| \|  \| MTFR \| 40239 \| 3229 \| 10441 \| -33 \| 135060 \| -43 \| 188969 \| -37 \| \| Adriatic Sea \| \|  \|  \|  \|  \|  \|  \|  \|  \| \|  \| REF \| 346900 \| 11802 \| 50053 \|  \| 884785 \|  \| 1293540 \|  \| \|  \| BAU \| 343854 \| 11665 \| 46745 \| -7 \| 876638 \| -1 \| 1278902 \| -1 \| \|  \| NUTR \| 346900 \| 9682 \| 40931 \| -18 \| 848085 \| -4 \| 1245597 \| -4 \| \|  \| MTFR \| 346900 \| 9682 \| 39632 \| -21 \| 534385 \| -40 \| 930598 \| -28 \| \| Western Med Sea \| \| \|  \|  \|  \|  \|  \|  \|  \| \|  \| REF \| 313139 \| 6087 \| 98436 \|  \| 1262171 \|  \| 1679833 \|  \| \|  \| BAU \| 310041 \| 6053 \| 96028 \| -2 \| 1249788 \| -1 \| 1661910 \| -1 \| \|  \| NUTR \| 313139 \| 5072 \| 81316 \| -17 \| 1224325 \| -3 \| 1623852 \| -3 \| \|  \| MTFR \| 313139 \| 5072 \| 65512 \| -33 \| 837689 \| -34 \| 1221412 \| -27 \| |
| --- | --- | --- | --- | --- | --- | --- | --- | --- | --- | --- | --- | --- | --- | --- | --- | --- | --- | --- | --- | --- | --- | --- | --- | --- | --- | --- | --- | --- | --- | --- | --- | --- | --- | --- | --- | --- | --- | --- | --- | --- | --- | --- | --- | --- | --- | --- | --- | --- | --- | --- | --- | --- | --- | --- | --- | --- | --- | --- | --- | --- | --- | --- | --- | --- | --- | --- | --- | --- | --- | --- | --- | --- | --- | --- | --- | --- | --- | --- | --- | --- | --- | --- | --- | --- | --- | --- | --- | --- | --- | --- | --- | --- | --- | --- | --- | --- | --- | --- | --- | --- | --- | --- | --- | --- | --- | --- | --- | --- | --- | --- | --- | --- | --- | --- | --- | --- | --- | --- | --- | --- | --- | --- | --- | --- | --- | --- | --- | --- | --- | --- | --- | --- | --- | --- | --- | --- | --- | --- | --- | --- | --- | --- | --- | --- | --- | --- | --- | --- | --- | --- | --- | --- | --- | --- | --- | --- | --- | --- | --- | --- | --- | --- | --- | --- | --- | --- | --- | --- | --- | --- | --- | --- | --- | --- | --- | --- | --- | --- | --- | --- | --- | --- | --- | --- | --- | --- | --- | --- | --- | --- | --- | --- | --- | --- | --- | --- | --- | --- | --- | --- | --- | --- | --- | --- | --- | --- | --- | --- | --- | --- | --- | --- | --- | --- | --- | --- | --- | --- | --- | --- | --- | --- | --- | --- | --- | --- | --- | --- | --- | --- | --- | --- | --- | --- | --- | --- | --- | --- | --- | --- | --- | --- | --- | --- | --- | --- | --- | --- | --- | --- | --- | --- | --- | --- | --- | --- | --- | --- | --- | --- | --- | --- | --- | --- | --- | --- | --- | --- | --- | --- | --- | --- | --- | --- | --- | --- | --- | --- | --- | --- | --- | --- | --- | --- | --- | --- | --- | --- | --- | --- | --- | --- | --- | --- | --- | --- | --- | --- | --- | --- | --- | --- | --- | --- | --- | --- | --- | --- | --- | --- | --- | --- | --- | --- | --- | --- | --- | --- | --- | --- | --- | --- | --- | --- | --- | --- | --- | --- | --- | --- | --- | --- | --- | --- | --- | --- | --- | --- | --- | --- | --- | --- | --- | --- | --- | --- | --- | --- | --- | --- | --- | --- | --- | --- | --- | --- | --- | --- | --- | --- | --- | --- | --- | --- | --- | --- | --- | --- | --- | --- | --- | --- | --- | --- | --- | --- | --- | --- | --- | --- | --- | --- | --- | --- | --- | --- | --- | --- | --- | --- | --- | --- | --- | --- | --- | --- | --- | --- | --- | --- | --- | --- | --- | --- | --- | --- | --- | --- | --- | --- | --- | --- | --- | --- | --- | --- | --- | --- | --- | --- | --- | --- | --- | --- | --- | --- | --- | --- | --- | --- | --- | --- | --- | --- | --- | --- | --- | --- | --- | --- | --- | --- | --- | --- | --- | --- | --- | --- | --- | --- | --- | --- | --- | --- | --- | --- | --- | --- | --- | --- | --- | --- | --- | --- | --- | --- | --- | --- | --- | --- | --- | --- | --- | --- | --- | --- | --- | --- | --- | --- |
|  |

Table S5.2 Phosphorus inland input per regional seas (per source and scenario).

| **Phosphorus inland input per regional seas (per source and scenario)** | | | | | | | | | |
| --- | --- | --- | --- | --- | --- | --- | --- | --- | --- |
|  | | **Background** | **Scattered dwellings** | **Point sources** |  | **Agriculture (Min, Man)** |  | **Total Input** |  |
|  | Scenario | (ton/y) | (ton/y) | (ton/y) | % of change | (ton/y) | % of change | (ton/y) | % of change |
| Baltic |  |  |  |  |  |  |  |  |  |
|  | REF | 24788 | 7790 | 12768 |  | 530411 |  | 575758 |  |
|  | BAU | 24788 | 7777 | 12095 | -5 | 521432 | -2 | 566092 | -2 |
|  | NUTR | 24788 | 7538 | 11268 | -12 | 507270 | -4 | 550864 | -4 |
|  | MTFR | 24788 | 7538 | 10329 | -19 | 442224 | -17 | 484879 | -16 |
| Greater North Sea | |  |  |  |  |  |  |  |  |
|  | REF | 14315 | 2995 | 32323 |  | 1033242 |  | 1082874 |  |
|  | BAU | 14315 | 2995 | 32300 | 0 | 1022322 | -1 | 1071932 | -1 |
|  | NUTR | 14315 | 2478 | 31229 | -3 | 738886 | -28 | 786908 | -27 |
|  | MTFR | 14315 | 2474 | 23205 | -28 | 938456 | -9 | 978450 | -10 |
| Celtic Sea |  |  |  |  |  |  |  |  |  |
|  | REF | 2929 | 301 | 9281 |  | 290097 |  | 302608 |  |
|  | BAU | 2929 | 301 | 8867 | -4 | 284694 | -2 | 296792 | -2 |
|  | NUTR | 2929 | 241 | 9153 | -1 | 279416 | -4 | 291739 | -4 |
|  | MTFR | 2929 | 213 | 4441 | -52 | 267602 | -8 | 275185 | -9 |
| Bay of Biscay and Iberian Coast | | |  |  |  |  |  |  |  |
|  | REF | 9909 | 1735 | 15884 |  | 566851 |  | 594379 |  |
|  | BAU | 9909 | 1720 | 14494 | -9 | 559545 | -1 | 585669 | -1 |
|  | NUTR | 9909 | 1714 | 14125 | -11 | 564704 | 0 | 590451 | -1 |
|  | MTFR | 9909 | 1710 | 7482 | -53 | 456045 | -20 | 475146 | -20 |
| Black Sea |  |  |  |  |  |  |  |  |  |
|  | REF | 16204 | 7965 | 28502 |  | 616570 |  | 669240 |  |
|  | BAU | 16204 | 7868 | 23064 | -19 | 609207 | -1 | 656342 | -2 |
|  | NUTR | 16204 | 7409 | 23618 | -17 | 576299 | -7 | 623529 | -7 |
|  | MTFR | 16204 | 7353 | 20102 | -29 | 564287 | -8 | 607946 | -9 |
| Aegean-Levantine Sea | | |  |  |  |  |  |  |  |
|  | REF | 5517 | 2897 | 12236 |  | 178358 |  | 199008 |  |
|  | BAU | 5517 | 2897 | 11747 | -4 | 177509 | 0 | 197670 | -1 |
|  | NUTR | 5517 | 2655 | 11760 | -4 | 177890 | 0 | 197821 | -1 |
|  | MTFR | 5517 | 2646 | 9845 | -20 | 159501 | -11 | 177509 | -11 |
| Ionian Sea and Central Med Sea | | | |  |  |  |  |  |  |
|  | REF | 1214 | 810 | 2441 |  | 37761 |  | 42226 |  |
|  | BAU | 1214 | 809 | 2346 | -4 | 36989 | -2 | 41357 | -2 |
|  | NUTR | 1214 | 525 | 2296 | -6 | 36806 | -3 | 40841 | -3 |
|  | MTFR | 1214 | 522 | 815 | -67 | 26250 | -30 | 28801 | -32 |
| Adriatic Sea | |  |  |  |  |  |  |  |  |
|  | REF | 3572 | 2321 | 7881 |  | 202626 |  | 216401 |  |
|  | BAU | 3572 | 2294 | 6741 | -14 | 200848 | -1 | 213455 | -1 |
|  | NUTR | 3572 | 1956 | 6128 | -22 | 186650 | -8 | 198306 | -8 |
|  | MTFR | 3572 | 1933 | 3906 | -50 | 154648 | -24 | 164059 | -24 |
| Western Med Sea | |  |  |  |  |  |  |  |  |
|  | REF | 6409 | 1150 | 17930 |  | 242760 |  | 268249 |  |
|  | BAU | 6409 | 1145 | 17354 | -3 | 240245 | -1 | 265152 | -1 |
|  | NUTR | 6409 | 986 | 16624 | -7 | 231508 | -5 | 255526 | -5 |
|  | MTFR | 6409 | 985 | 6863 | -62 | 179053 | -26 | 193310 | -28 |

Table S5.3 Nitrogen inland input in EU28 under REF and MTFR scenarios.

| **Nitrogen inland input in EU28 countries (per source and scenario MTFR)** | | | | | | | | | | |
| --- | --- | --- | --- | --- | --- | --- | --- | --- | --- | --- |
|  | **Scenario REF (ton)** | | | | | **Changes in scenario MTFR compared to scenario REF (%)** | | | | |
| **Country** | Atmospheric deposition | Scattered dwellings | Point sources | Agriculture | Total input | Atmospheric deposition | Scattered dwellings | Point sources | Agriculture | Total input |
| **AT** | 107290 | 0 | 15487 | 256670 | 379447 | 0 | -40 | 0 | -3 | -2 |
| **BE** | 52005 | 2502 | 11528 | 382658 | 448693 | 0 | 0 | -17 | -31 | -26 |
| **BG** | 74982 | 1159 | 10761 | 293765 | 380667 | 0 | 0 | -46 | -22 | -19 |
| **CY** | 2959 | 673 | 1099 | 15262 | 19993 | 0 | -2 | -27 | -26 | -22 |
| **CZ** | 95376 | 4822 | 7546 | 449503 | 557248 | 0 | -14 | -13 | -51 | -41 |
| **DE** | 582254 | 3259 | 83122 | 2658311 | 3326944 | 0 | -64 | -1 | -24 | -19 |
| **DK** | 49490 | 0 | 9113 | 440232 | 498835 | 0 | 0 | -1 | -25 | -22 |
| **EE** | 27418 | 1 | 1052 | 84117 | 112588 | 0 | -40 | -2 | -24 | -18 |
| **ES** | 241078 | 228 | 73957 | 1697343 | 2012607 | 0 | -47 | -34 | -43 | -37 |
| **FI** | 103108 | 2599 | 7931 | 222687 | 336325 | 0 | 0 | -1 | -29 | -19 |
| **FR** | 603176 | 16655 | 71311 | 3583686 | 4274828 | 0 | 0 | -14 | -9 | -8 |
| **GB** | 194772 | 744 | 109117 | 1761634 | 2066267 | 0 | -52 | -35 | -33 | -30 |
| **GR** | 78778 | 5172 | 8810 | 372337 | 465097 | 0 | -25 | -4 | -48 | -39 |
| **HR** | 57536 | 576 | 8780 | 166449 | 233341 | 0 | -64 | -67 | -63 | -47 |
| **HU** | 84353 | 3602 | 8389 | 442588 | 538932 | 0 | -35 | -19 | -37 | -31 |
| **IE** | 50028 | 1108 | 7810 | 727191 | 786137 | 0 | -40 | -50 | 0 | -1 |
| **IT** | 373198 | 9510 | 91189 | 1187167 | 1661064 | 0 | -47 | -28 | -43 | -32 |
| **LT** | 58698 | 2548 | 2225 | 241232 | 304703 | 0 | -10 | -1 | -19 | -15 |
| **LU** | 4045 | 40 | 503 | 26626 | 31214 | 0 | -18 | -8 | -45 | -39 |
| **LV** | 45071 | 1545 | 1345 | 97957 | 145917 | 0 | -8 | -24 | -19 | -13 |
| **MT** | 109 | 0 | 655 | 2724 | 3488 | 0 | 0 | -3 | -45 | -36 |
| **NL** | 74042 | 263 | 18159 | 590486 | 682951 | 0 | 0 | -1 | -25 | -22 |
| **PL** | 352123 | 18643 | 39566 | 1594469 | 2004801 | 0 | -2 | -16 | -28 | -23 |
| **PT** | 33459 | 664 | 20319 | 186077 | 240520 | 0 | 0 | -41 | -35 | -31 |
| **RO** | 187531 | 1778 | 51876 | 784563 | 1025747 | 0 | -5 | -53 | -11 | -11 |
| **SE** | 158667 | 0 | 12611 | 298413 | 469692 | 0 | 0 | 0 | -14 | -9 |
| **SI** | 27698 | 2160 | 2362 | 42294 | 74515 | 0 | -6 | -49 | -26 | -16 |
| **SK** | 47480 | 4453 | 4133 | 161768 | 217834 | 0 | -16 | -21 | -26 | -20 |
| **Total EU28** | 3766724 | 84702 | 680757 | 18768211 | 23300394 | 0 | -15 | -24 | -25 | -21 |

Table S5.4 Phosphorus inland input in EU28 under REF and MTFR scenarios.

| **Phosphorus inland input in EU28 countries (per source and scenario MTFR)** | | | | | | | | | | | | | |
| --- | --- | --- | --- | --- | --- | --- | --- | --- | --- | --- | --- | --- | --- |
|  | | **Scenario REF (ton)** | | | | | | **Changes in scenario MTFR compared to scenario REF (%)** | | | | | |
| **Country** | Background | | Scattered dwellings | Point sources | Agriculture | Total input | Background | | Scattered dwellings | Point sources | Agriculture | Total input |  |
| **AT** | 1258 | | 0 | 1265 | 61169 | 63692 | 0 | | -42 | -1 | -2 | -2 |  |
| **BE** | 461 | | 380 | 1480 | 87678 | 89999 | 0 | | 0 | -25 | -7 | -7 |  |
| **BG** | 1667 | | 209 | 1623 | 33197 | 36695 | 0 | | 0 | -67 | -16 | -17 |  |
| **CY** | 139 | | 217 | 313 | 4132 | 4801 | 0 | | -2 | -42 | -21 | -21 |  |
| **CZ** | 1184 | | 1043 | 1032 | 47709 | 50968 | 0 | | -16 | -30 | -26 | -25 |  |
| **DE** | 5360 | | 513 | 7198 | 445985 | 459056 | 0 | | -74 | -7 | -10 | -10 |  |
| **DK** | 647 | | 0 | 883 | 64753 | 66283 | 0 | | 0 | -1 | -5 | -5 |  |
| **EE** | 682 | | 0 | 113 | 10571 | 11366 | 0 | | -42 | -3 | -20 | -19 |  |
| **ES** | 7470 | | 52 | 15338 | 370825 | 393686 | 0 | | -52 | -59 | -35 | -35 |  |
| **FI** | 5090 | | 416 | 523 | 31831 | 37860 | 0 | | 0 | -3 | -15 | -12 |  |
| **FR** | 8234 | | 3588 | 11947 | 591610 | 615379 | 0 | | 0 | -43 | -5 | -6 |  |
| **GB** | 3665 | | 165 | 20048 | 276421 | 300299 | 0 | | -59 | -56 | -18 | -20 |  |
| **GR** | 1974 | | 1075 | 2936 | 57175 | 63161 | 0 | | -27 | -67 | -39 | -39 |  |
| **HR** | 845 | | 179 | 2631 | 23647 | 27303 | 0 | | -74 | -83 | -46 | -49 |  |
| **HU** | 1389 | | 761 | 1200 | 58865 | 62215 | 0 | | -38 | -42 | -23 | -23 |  |
| **IE** | 1047 | | 161 | 1035 | 141523 | 143767 | 0 | | -46 | -70 | 0 | -1 |  |
| **IT** | 4502 | | 1425 | 12642 | 250632 | 269200 | 0 | | -52 | -59 | -26 | -27 |  |
| **LT** | 985 | | 435 | 208 | 33332 | 34960 | 0 | | -12 | -2 | -15 | -14 |  |
| **LU** | 39 | | 6 | 45 | 4910 | 4999 | 0 | | -21 | -17 | -18 | -18 |  |
| **LV** | 964 | | 286 | 176 | 16800 | 18226 | 0 | | -9 | -39 | -17 | -16 |  |
| **MT** | 5 | | 0 | 274 | 646 | 924 | 0 | | 0 | -50 | -21 | -30 |  |
| **NL** | 519 | | 37 | 1679 | 116145 | 118380 | 0 | | 0 | -6 | -4 | -4 |  |
| **PL** | 4678 | | 5277 | 6790 | 319049 | 335795 | 0 | | -2 | -34 | -20 | -20 |  |
| **PT** | 1329 | | 150 | 4477 | 43652 | 49608 | 0 | | 0 | -55 | -29 | -30 |  |
| **RO** | 3583 | | 290 | 8341 | 127108 | 139323 | 0 | | -5 | -65 | -7 | -11 |  |
| **SE** | 6743 | | 0 | 1043 | 42355 | 50141 | 0 | | 0 | 0 | -7 | -6 |  |
| **SI** | 305 | | 346 | 356 | 12801 | 13807 | 0 | | -7 | -64 | -15 | -16 |  |
| **SK** | 730 | | 888 | 540 | 15396 | 17555 | 0 | | -18 | -46 | -16 | -16 |  |
| **Total EU28** | 65494 | | 17898 | 106137 | 3289917 | 3479447 | 0 | | -14 | -49 | -16 | -16 |  |

## S6. Nutrient loads to European regional seas under different scenarios

Table S6.1 Nitrogen load at sea outlets (per source and scenario) estimated by the model GREEN.

| **Nitrogen load at sea outlets (per source and scenario)** | | | | | | | | | |
| --- | --- | --- | --- | --- | --- | --- | --- | --- | --- |
|  | | **Atmospheric deposition** | **Scattered dwellings** | **Point sources** |  | **Agriculture (Min, Man, BNF)** |  | **Total load** |  |
|  | Scenario | (ton/y) | (ton/y) | (ton/y) | % of change | (ton/y) | % of change | (ton/y) | % of change |
| Baltic |  |  |  |  |  |  |  |  |  |
|  | REF | 203992 | 20171 | 83561 |  | 217699 |  | 525423 |  |
|  | BAU | 200051 | 20145 | 81745 | -2 | 213669 | -2 | 515608 | -2 |
|  | NUTR | 203963 | 19530 | 79532 | -5 | 213140 | -2 | 516165 | -2 |
|  | MTFR | 203962 | 19530 | 77214 | -8 | 171710 | -21 | 472416 | -10 |
| Greater North Sea | | |  |  |  |  |  |  |  |
|  | REF | 226159 | 9815 | 236788 |  | 655866 |  | 1128627 |  |
|  | BAU | 223834 | 9815 | 236746 | 0 | 648476 | -1 | 1118870 | -1 |
|  | NUTR | 226156 | 8137 | 227692 | -4 | 564141 | -14 | 1026126 | -9 |
|  | MTFR | 226156 | 8137 | 206589 | -13 | 509292 | -22 | 950173 | -16 |
| Celtic Sea | |  |  |  |  |  |  |  |  |
|  | REF | 36655 | 1149 | 54908 |  | 324813 |  | 417525 |  |
|  | BAU | 35968 | 1149 | 52503 | -4 | 318664 | -2 | 408284 | -2 |
|  | NUTR | 36654 | 818 | 48631 | -11 | 316696 | -2 | 402798 | -4 |
|  | MTFR | 36654 | 818 | 37382 | -32 | 265481 | -18 | 340335 | -18 |
| Bay of Biscay and Iberian Coast | | | |  |  |  |  |  |  |
|  | REF | 86320 | 5132 | 75714 |  | 209743 |  | 376908 |  |
|  | BAU | 85332 | 5092 | 71677 | -5 | 207585 | -1 | 369686 | -2 |
|  | NUTR | 86318 | 5066 | 62208 | -18 | 209225 | 0 | 362817 | -4 |
|  | MTFR | 86317 | 5066 | 52697 | -30 | 178043 | -15 | 322123 | -15 |
| Black Sea | |  |  |  |  |  |  |  |  |
|  | REF | 163158 | 21202 | 149583 |  | 191089 |  | 525033 |  |
|  | BAU | 160717 | 20937 | 125850 | -16 | 188359 | -1 | 495863 | -6 |
|  | NUTR | 163157 | 19873 | 118845 | -21 | 183064 | -4 | 484939 | -8 |
|  | MTFR | 163156 | 19873 | 117262 | -22 | 162536 | -15 | 462828 | -12 |
| Aegean-Levantine Sea | | |  |  |  |  |  |  |  |
|  | REF | 50016 | 9016 | 52978 |  | 81797 |  | 193807 |  |
|  | BAU | 49748 | 9016 | 51103 | -4 | 81435 | 0 | 191303 | -1 |
|  | NUTR | 50010 | 8285 | 50782 | -4 | 81657 | 0 | 190735 | -2 |
|  | MTFR | 50010 | 8285 | 50619 | -4 | 73137 | -11 | 182051 | -6 |
| Ionian Sea and Central Med Sea | | | |  |  |  |  |  |  |
|  | REF | 9310 | 3258 | 15343 |  | 22389 |  | 50300 |  |
|  | BAU | 9124 | 3254 | 14754 | -4 | 21928 | -2 | 49061 | -2 |
|  | NUTR | 9308 | 2125 | 11112 | -28 | 22122 | -1 | 44667 | -11 |
|  | MTFR | 9305 | 2125 | 10340 | -33 | 12504 | -44 | 34274 | -32 |
| Adriatic Sea | |  |  |  |  |  |  |  |  |
|  | REF | 81602 | 7588 | 48586 |  | 95888 |  | 233665 |  |
|  | BAU | 80909 | 7497 | 45317 | -7 | 95150 | -1 | 228873 | -2 |
|  | NUTR | 81598 | 6196 | 39670 | -18 | 91711 | -4 | 219174 | -6 |
|  | MTFR | 81540 | 6196 | 38365 | -21 | 60995 | -36 | 187096 | -20 |
| Western Med Sea | | |  |  |  |  |  |  |  |
|  | REF | 74916 | 3956 | 96524 |  | 75350 |  | 250746 |  |
|  | BAU | 74189 | 3933 | 94154 | -2 | 74507 | -1 | 246783 | -2 |
|  | NUTR | 74913 | 3281 | 79612 | -18 | 74324 | -1 | 232131 | -7 |
|  | MTFR | 74907 | 3281 | 64084 | -34 | 58814 | -22 | 201086 | -20 |

Table S6.2 Phosphorus load at sea outlets (per source and scenario) estimated by the model GREEN.

| **Phosphorus load at sea outlets (per source and scenario)** | | | | | | | | | |
| --- | --- | --- | --- | --- | --- | --- | --- | --- | --- |
|  | | **Background sources** | **Scattered dwellings** | **Point sources** |  | **Agriculture (Min, Man)** |  | **Total load** |  |
|  | Scenario | (ton/y) | (ton/y) | (ton/y) | % of change | (ton/y) | % of change | (ton/y) | % of change |
| Baltic |  |  |  |  |  |  |  |  |  |
|  | REF | 12342 | 4473 | 10733 |  | 6317 |  | 33865 |  |
|  | BAU | 12341 | 4465 | 10191 | -5 | 6178 | -2 | 33175 | -2 |
|  | NUTR | 12341 | 4320 | 9525 | -11 | 5987 | -5 | 32174 | -5 |
|  | MTFR | 12341 | 4320 | 8772 | -18 | 5321 | -16 | 30755 | -9 |
| Greater North Sea | |  |  |  |  |  |  |  |  |
|  | REF | 5685 | 1809 | 30196 |  | 28389 |  | 66079 |  |
|  | BAU | 5685 | 1809 | 30176 | 0 | 28062 | -1 | 65731 | -1 |
|  | NUTR | 5685 | 1485 | 29186 | -3 | 20438 | -28 | 56793 | -14 |
|  | MTFR | 5685 | 1482 | 21449 | -29 | 25879 | -9 | 54495 | -18 |
| Celtic Sea |  |  |  |  |  |  |  |  |  |
|  | REF | 1085 | 210 | 9215 |  | 21487 |  | 31997 |  |
|  | BAU | 1085 | 210 | 8802 | -4 | 21078 | -2 | 31175 | -3 |
|  | NUTR | 1085 | 168 | 9089 | -1 | 20764 | -3 | 31105 | -3 |
|  | MTFR | 1085 | 148 | 4405 | -52 | 19461 | -9 | 25099 | -22 |
| Bay of Biscay and Iberian Coast | | |  |  |  |  |  |  |  |
|  | REF | 3774 | 1123 | 14598 |  | 9264 |  | 28759 |  |
|  | BAU | 3774 | 1113 | 13381 | -8 | 9163 | -1 | 27431 | -5 |
|  | NUTR | 3774 | 1108 | 13020 | -11 | 9225 | 0 | 27127 | -6 |
|  | MTFR | 3774 | 1106 | 6775 | -54 | 8067 | -13 | 19722 | -31 |
| Black Sea |  |  |  |  |  |  |  |  |  |
|  | REF | 4551 | 3487 | 19398 |  | 5892 |  | 33328 |  |
|  | BAU | 4551 | 3451 | 15593 | -20 | 5775 | -2 | 29370 | -12 |
|  | NUTR | 4551 | 3287 | 15972 | -18 | 5360 | -9 | 29170 | -12 |
|  | MTFR | 4551 | 3266 | 13704 | -29 | 5475 | -7 | 26995 | -19 |
| Aegean-Levantine Sea | | |  |  |  |  |  |  |  |
|  | REF | 2959 | 1935 | 11653 |  | 4384 |  | 20931 |  |
|  | BAU | 2959 | 1935 | 11206 | -4 | 4374 | 0 | 20474 | -2 |
|  | NUTR | 2959 | 1766 | 11219 | -4 | 4380 | 0 | 20323 | -3 |
|  | MTFR | 2959 | 1759 | 9311 | -20 | 4186 | -5 | 18215 | -13 |
| Ionian Sea and Central Med Sea | | | |  |  |  |  |  |  |
|  | REF | 582 | 569 | 2402 |  | 1021 |  | 4574 |  |
|  | BAU | 582 | 568 | 2308 | -4 | 1001 | -2 | 4459 | -3 |
|  | NUTR | 582 | 368 | 2260 | -6 | 998 | -2 | 4208 | -8 |
|  | MTFR | 582 | 366 | 802 | -67 | 683 | -33 | 2432 | -47 |
| Adriatic Sea | |  |  |  |  |  |  |  |  |
|  | REF | 1776 | 1563 | 7369 |  | 6650 |  | 17359 |  |
|  | BAU | 1776 | 1544 | 6282 | -15 | 6604 | -1 | 16207 | -7 |
|  | NUTR | 1776 | 1312 | 5769 | -22 | 6170 | -7 | 15028 | -13 |
|  | MTFR | 1776 | 1295 | 3599 | -51 | 5274 | -21 | 11945 | -31 |
| Western Med Sea | |  |  |  |  |  |  |  |  |
|  | REF | 3270 | 759 | 17126 |  | 3514 |  | 24669 |  |
|  | BAU | 3270 | 755 | 16576 | -3 | 3473 | -1 | 24075 | -2 |
|  | NUTR | 3270 | 642 | 15880 | -7 | 3455 | -2 | 23248 | -6 |
|  | MTFR | 3270 | 642 | 6515 | -62 | 3043 | -13 | 13471 | -45 |

## S7. Nutrient concentration in European rivers under different scenarios

Table S7.1 Statistics on the distribution of nitrogen (mgN/l) and phosphorus (mgP/l) concentration and N:P ratio (mgN/mgP) at all catchments outlets, estimated by the model GREEN under different scenarios. (Trimmed: trimmed mean, mean computed dropping the 10% top and bottom fraction of the data).

Nitrogen all catchments

| Scenario | n | mean | sd | median | trimmed | Q0.05 | Q0.1 | Q0.25 | Q0.75 | Q0.9 | Q0.95 |
| --- | --- | --- | --- | --- | --- | --- | --- | --- | --- | --- | --- |
| BAU | 845314 | 2.49 | 18.11 | 1.05 | 1.40 | 0.08 | 0.15 | 0.47 | 2.32 | 4.69 | 7.19 |
| NUTR | 845314 | 2.45 | 17.71 | 1.06 | 1.40 | 0.08 | 0.15 | 0.48 | 2.32 | 4.63 | 7.01 |
| MTFR | 845347 | 2.26 | 21.53 | 0.99 | 1.29 | 0.08 | 0.15 | 0.46 | 2.11 | 4.21 | 6.32 |
| REF | 845312 | 2.54 | 18.19 | 1.07 | 1.42 | 0.08 | 0.15 | 0.48 | 2.36 | 4.77 | 7.33 |

Phosphorus all catchments

| Scenario | n | mean | sd | median | trimmed | Q0.05 | Q0.1 | Q0.25 | Q0.75 | Q0.9 | Q0.95 |
| --- | --- | --- | --- | --- | --- | --- | --- | --- | --- | --- | --- |
| BAU | 845314 | 0.16 | 2.16 | 0.06 | 0.07 | 0.02 | 0.02 | 0.03 | 0.12 | 0.25 | 0.42 |
| NUTR | 845314 | 0.15 | 2.05 | 0.06 | 0.07 | 0.02 | 0.02 | 0.03 | 0.11 | 0.24 | 0.40 |
| MTFR | 845347 | 0.14 | 2.51 | 0.05 | 0.07 | 0.02 | 0.02 | 0.03 | 0.11 | 0.22 | 0.37 |
| REF | 845312 | 0.17 | 2.19 | 0.06 | 0.08 | 0.02 | 0.02 | 0.03 | 0.12 | 0.25 | 0.43 |

N:P ratio

| Scenario | n | mean | sd | median | trimmed | Q0.05 | Q0.1 | Q0.25 | Q0.75 | Q0.9 | Q0.95 |
| --- | --- | --- | --- | --- | --- | --- | --- | --- | --- | --- | --- |
| BAU | 845314 | 18.95 | 16.68 | 16.64 | 17.49 | 3.08 | 5.35 | 11.43 | 23.82 | 33.35 | 40.99 |
| NUTR | 845314 | 19.39 | 16.99 | 16.90 | 17.85 | 3.11 | 5.35 | 11.57 | 24.40 | 34.68 | 42.56 |
| MTFR | 845347 | 18.79 | 16.71 | 16.58 | 17.37 | 3.12 | 5.48 | 11.55 | 23.35 | 32.73 | 40.31 |
| REF | 845312 | 19.06 | 16.71 | 16.70 | 17.59 | 3.12 | 5.39 | 11.45 | 23.98 | 33.70 | 41.23 |

Table S7.2 Statistics on the distribution of nitrogen (mgN/l) and phosphorus (mgP/l) concentration and N:P ratio (mgN/mgP) at the river basins outlets, estimated by the model GREEN under different scenarios. (Trimmed: trimmed mean, mean computed dropping the 10% top and bottom fraction of the data).

| Scenario | n | mean | sd | median | trimmed | Q0.05 | Q0.1 | Q0.25 | Q0.75 | Q0.9 | Q0.95 |
| --- | --- | --- | --- | --- | --- | --- | --- | --- | --- | --- | --- |
| BAU | 4148 | 4.50 | 18.06 | 1.16 | 1.63 | 0.04 | 0.06 | 0.25 | 2.98 | 6.24 | 15.26 |
| NUTR | 4148 | 4.09 | 15.55 | 1.15 | 1.56 | 0.04 | 0.06 | 0.25 | 2.82 | 5.88 | 14.25 |
| MTFR | 4148 | 3.77 | 14.94 | 0.99 | 1.38 | 0.04 | 0.06 | 0.24 | 2.43 | 5.30 | 12.98 |
| REF | 4148 | 4.57 | 18.25 | 1.19 | 1.66 | 0.04 | 0.06 | 0.25 | 3.07 | 6.42 | 15.61 |

Nitrogen Sea Outlets

Phosphorus Sea Outlets

| Scenario | n | mean | sd | median | trimmed | Q0.05 | Q0.1 | Q0.25 | Q0.75 | Q0.9 | Q0.95 |
| --- | --- | --- | --- | --- | --- | --- | --- | --- | --- | --- | --- |
| BAU | 4148 | 0.37 | 1.71 | 0.07 | 0.11 | 0.01 | 0.01 | 0.03 | 0.20 | 0.52 | 1.28 |
| NUTR | 4148 | 0.33 | 1.55 | 0.07 | 0.11 | 0.01 | 0.01 | 0.03 | 0.18 | 0.46 | 1.17 |
| MTFR | 4148 | 0.29 | 1.42 | 0.06 | 0.10 | 0.01 | 0.01 | 0.03 | 0.17 | 0.38 | 0.93 |
| REF | 4148 | 0.38 | 1.72 | 0.07 | 0.12 | 0.01 | 0.01 | 0.03 | 0.20 | 0.53 | 1.36 |

N:P Sea Outlets

| Scenario | n | mean | sd | median | trimmed | Q0.05 | Q0.1 | Q0.25 | Q0.75 | Q0.9 | Q0.95 |
| --- | --- | --- | --- | --- | --- | --- | --- | --- | --- | --- | --- |
| BAU | 4148 | 14.00 | 10.03 | 12.97 | 13.08 | 1.75 | 3.61 | 8.11 | 18.11 | 23.97 | 28.75 |
| NUTR | 4148 | 14.75 | 11.65 | 13.11 | 13.26 | 1.75 | 3.51 | 7.99 | 18.59 | 25.56 | 34.31 |
| MTFR | 4148 | 13.79 | 9.40 | 13.25 | 13.12 | 1.75 | 3.64 | 8.62 | 17.62 | 22.26 | 26.58 |
| REF | 4148 | 14.04 | 10.07 | 12.98 | 13.12 | 1.75 | 3.61 | 8.14 | 18.25 | 24.05 | 28.93 |

Figure S7.1 Distribution of nitrogen and phosphorus concentration and N:P ratio at the sea outlets (right) and at all catchments outlets (left), estimated by the model GREEN under different scenarios.

| Europe - All catchments (left)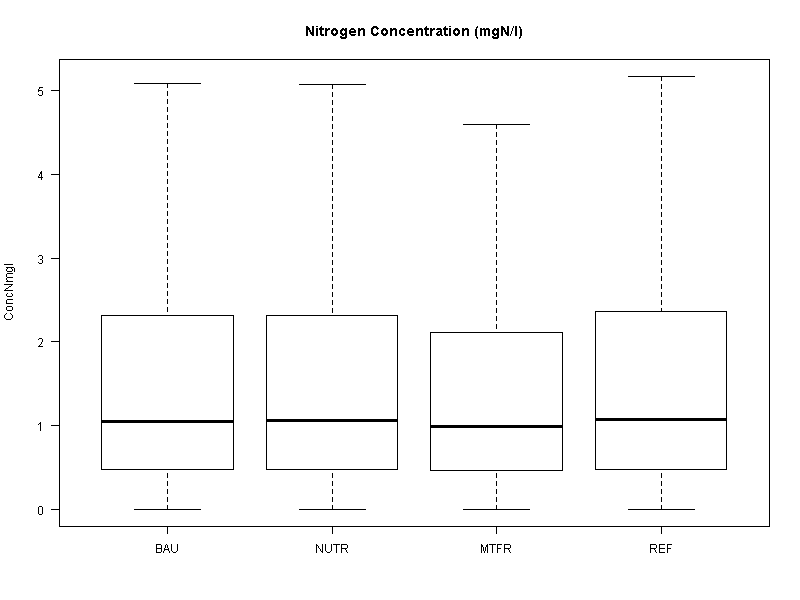 | Europe - Sea outlets (right)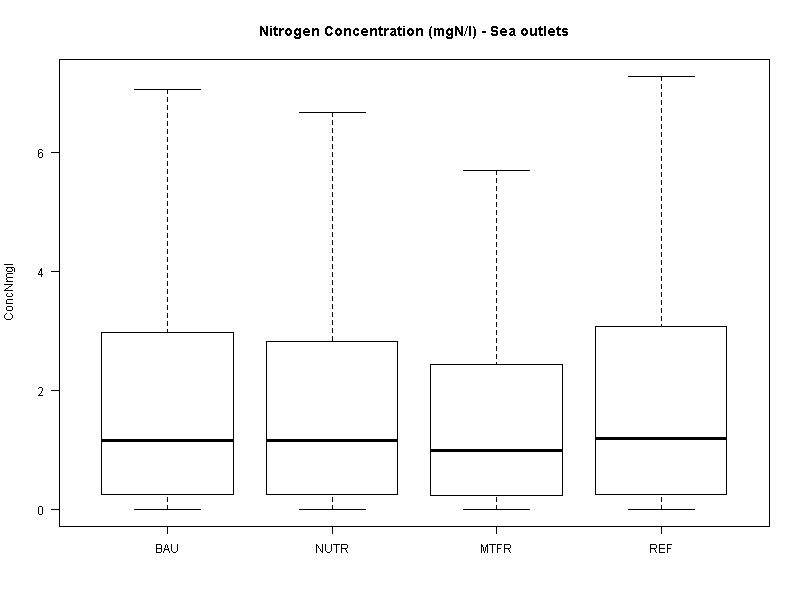 |
| --- | --- |
| 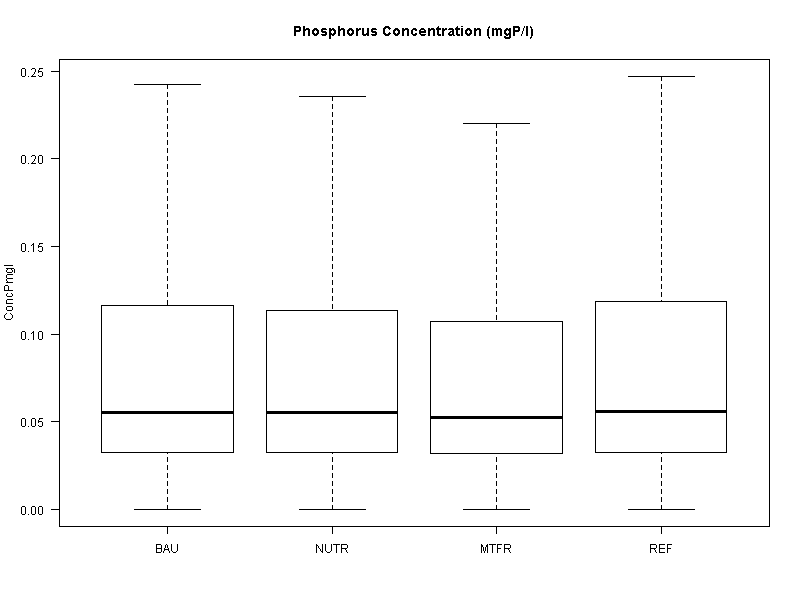 | 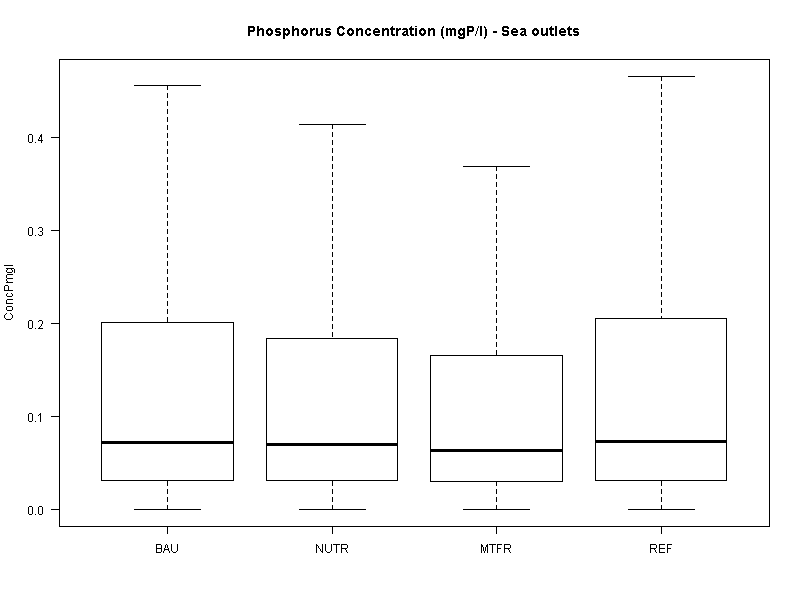 |
| 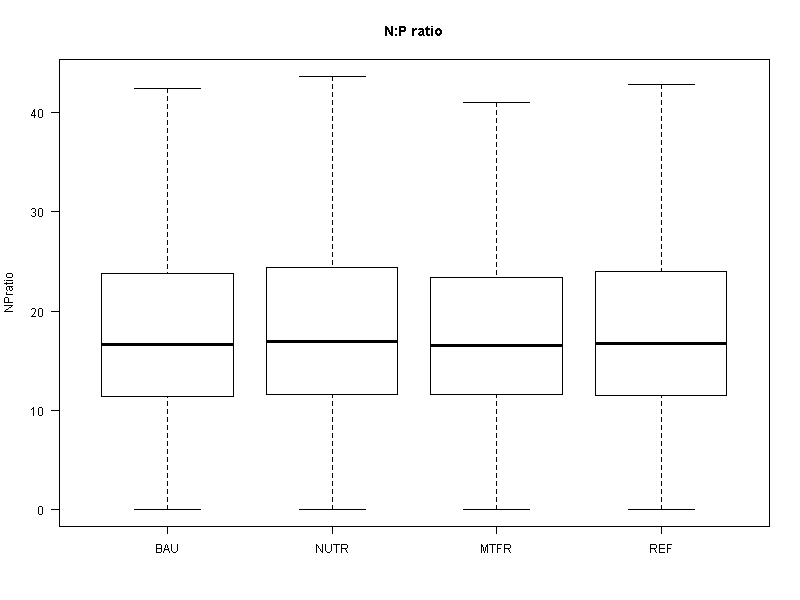 | 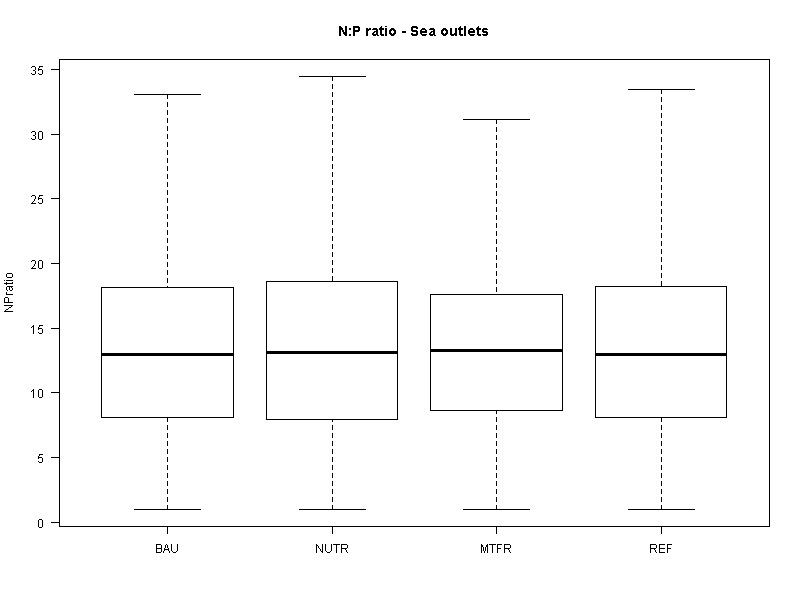 |
